# Regulation of Iron Storage by CsrA Supports Exponential Growth of Escherichia coli

**DOI:** 10.1128/mBio.01034-19

**Published:** 2019-08-06

**Authors:** Christine Pourciau, Archana Pannuri, Anastasia Potts, Helen Yakhnin, Paul Babitzke, Tony Romeo

**Affiliations:** a Department of Microbiology and Cell Science, Institute of Food and Agricultural Sciences, University of Florida, Gainesville, Florida, USA; b Department of Biochemistry and Molecular Biology, Center for RNA Molecular Biology, The Pennsylvania State University, University Park, Pennsylvania, USA; Princeton University

**Keywords:** CsrA, CsrB, CsrC, RNA binding proteins, bacterial growth, ferritin, iron homeostasis, iron storage proteins, oxidative stress, posttranscriptional regulation, sRNAs, stress responses

## Abstract

The global regulatory protein CsrA coordinates gene expression in response to physiological cues reflecting cellular stress and nutrition. CsrA binding to the 5′ segments of mRNA targets affects their translation, RNA stability, and/or transcript elongation. Recent studies identified probable mRNA targets of CsrA that are involved in iron uptake and storage in Escherichia coli, suggesting an unexplored role for CsrA in regulating iron homeostasis. Here, we assessed the impact of CsrA on iron-related gene expression, cellular iron, and growth under various iron levels. We investigated five new targets of CsrA regulation, including the genes for 4 ferritin or ferritin-like iron storage proteins (ISPs) and the stress-inducible Fe-S repair protein, SufA. CsrA bound with high affinity and specificity to *ftnB, bfr*, and *dps* mRNAs and inhibited their translation, while it modestly activated *ftnA* expression. Furthermore, CsrA was found to regulate cellular iron levels and support growth by repressing the expression of genes for ISPs, most importantly, ferritin B (FtnB) and bacterioferritin (Bfr). Iron starvation did not substantially affect cellular levels of CsrA or its small RNA (sRNA) antagonists, CsrB and CsrC. *csrA* disruption led to increased resistance to the lethal effects of H_2_O_2_ during exponential growth, consistent with a regulatory role in oxidative stress resistance. We propose that during exponential growth and under minimal stress, CsrA represses the deleterious expression of the ISPs that function under oxidative stress and stationary-phase conditions (FtnB, Bfr, and Dps), thus ensuring that cellular iron is available to processes that are required for growth.

## Abstract (precis)

Iron is an essential micronutrient for nearly all living organisms but is toxic in excess. Consequently, the maintenance of iron homeostasis is a critical biological process, and the genes involved in this function are tightly regulated. Here, we explored a new role for the bacterial RNA binding protein CsrA in the regulation of iron homeostasis. CsrA was shown to be a key regulator of iron storage genes in Escherichia coli, with consequential effects on cellular iron levels and growth. Our findings establish a model in which robust CsrA activity during the exponential phase of growth leads to repression of genes whose products sequester iron or divert it to unnecessary stress response processes. In so doing, CsrA supports E. coli growth under iron-limiting laboratory conditions and may promote fitness in the competitive iron-limited environment of the host large intestine.

## INTRODUCTION

Bacteria such as Escherichia coli have evolved complex and efficient global regulatory systems that enable them to recognize and adapt to changing environmental conditions, thus supporting their growth, survival, competition, and host-microbe interactions. The carbon storage regulatory system (Csr) is one such system and plays a critical a role in controlling numerous important cellular processes, including central carbon metabolism (1–3), stress response systems (4–6), biofilm formation (7), motility (8), quorum sensing (9), and virulence properties of pathogens (10). The Csr system is widely distributed among bacteria, with homologous regulatory factors sometimes referred to as Rsm (repressor of secondary metabolites) (11, 12).

The key component of the Csr system is CsrA, a homodimeric, sequence-specific RNA-binding protein, encoded by *csrA* (13–15). In general, CsrA represses the expression of genes associated with stress responses and stationary-phase growth (4, 16), while activating the expression of genes that support exponential growth (16, 17). CsrA binds to mRNA targets at sites containing a GGA motif surrounded by semiconserved sequences, with the GGA often located in the single-stranded loop of a hairpin (18, 19). The binding of CsrA to sites located in the 5′ untranslated region (5′ UTR) or the early coding region can result in altered translation (20–23), altered RNA stability (8, 24), and/or changes in RNA secondary structure that affect Rho-dependent transcription termination (25).

CsrA itself is subject to multifaceted regulation (16). While *csrA* gene expression is regulated transcriptionally and posttranscriptionally (26), CsrA activity is controlled by the small RNAs (sRNAs) CsrB and CsrC. These sRNAs contain multiple high-affinity binding sites that mimic those of target mRNAs, allowing CsrB/C to bind to and sequester many CsrA dimers (27). CsrB and CsrC transcription is activated by amino acid limitation or other stresses via ppGpp/DksA (4), metabolic end products, e.g., acetate and formate, via BarA-UvrY (12, 28), and extracytoplasmic stress via σ^E^ (6). Their RNase E-mediated turnover is triggered by glucose, via interaction of EIIA^glc^ with CsrD (29–31). Thus, CsrB/C sRNAs accumulate and sequester CsrA when preferred carbon resources have been expended, amino acids are limiting, metabolic end products have accumulated, and/or cells experience extracellular stress. Antagonism of CsrA by CsrB/C promotes the transition from glycolytic metabolism and active growth to gluconeogenesis, glycogen biosynthesis, and the formation of a stress-resistant phenotype (30).

A recent transcriptomics analysis in E. coli revealed that CsrA affects the expression of numerous genes involved in iron uptake and metabolism and confirmed its effects on levels of four such mRNAs (19). Several of the genes were of particular interest due to their importance in iron homeostasis and/or the large magnitude of CsrA effects (19). These effects of CsrA were suspected of occurring indirectly, as they were not confirmed by *in vivo* binding of CsrA to the mRNAs using cross-linking immunoprecipitation sequencing (CLIP-seq). However, CLIP-seq analyses have constraints, sources of bias (32), and are limited in sensitivity (33), potentially generating false-negative results.

Iron is an essential micronutrient for nearly all living organisms and required for fundamental processes such as respiration, central metabolism, genetic regulation, and DNA repair (34). It is the most common transition metal found in proteins, typically within heme or Fe-S prosthetic groups (35, 36). Although iron is abundant in many environments, ferric iron (Fe^3+^) predominates under aerobic conditions at neutral pH. Fe^3+^ has poor aqueous solubility and exists as insoluble salts and oxides or bound to host iron-binding proteins (37). Soluble ferrous iron (Fe^2+^) is biologically available but is rapidly oxidized to ferric iron at neutral or higher pH under aerobic conditions (35). Consequently, microbes have evolved high-affinity acquisition systems to scavenge iron (38). An added complication is that Fe^2+^ can be cytotoxic due to its role in producing damaging hydroxyl radicals (^·^OH) from H_2_O_2_ via the Fenton reaction. Thus, free intracellular iron levels are tightly controlled (34, 39). Additionally, iron availability can serve as an important cue regarding the environment of the bacterium, affecting virulence processes in many pathogens (38, 40).

Intracellular iron storage proteins (ISPs) sequester iron, providing iron reserves and protection against toxicity (35). Three related classes of bacterial ISPs exist: archetypal ferritins, heme-containing bacterioferritins, and Dps proteins (DNA protection during starvation). Each of these proteins is composed of identical subunits that form a roughly spherical shell surrounding a central cavity that acts as an iron storage reservoir (34). Ferritins and bacterioferritins are composed of 24 subunits and can accommodate 2,000 to 3,000 iron ions; Dps proteins have 12 subunits and can store ∼500 iron ions (34). A key feature of ISPs is the ferroxidase center, which binds two ferrous ions and oxidizes them using molecular oxygen or H_2_O_2_, forming a diferric intermediate that migrates to the central core for storage (35). Bacteria often possess multiple ferritin or bacterioferritin genes (34). E. coli expresses two ferritins (FtnA and FtnB), one bacterioferritin (Bfr), and one Dps.

Assembly of the iron-sulfur (Fe-S) clusters that serve as cofactors for enzymes is a crucial biological function, performed by Fe-S biogenesis systems (41, 42). In E. coli, most Fe-S cluster formation under nonstress conditions is by the “housekeeping” Isc pathway, encoded by the *iscRSUA-hscBA-fd*x (*isc*) operon (42, 43). Alternatively, the Suf pathway, encoded by the *sufABCDSE* (*suf*) operon, is more robust than Isc against oxidative damage and is favored for Fe-S assembly during oxidative and nitrosative stress or when iron is limiting (44–47). The coordinated regulation of these pathways accommodates the changing requirements for Fe-S cluster biogenesis, which fluctuate with growth conditions (43, 48).

In E. coli and most bacteria, the DNA-binding ferric uptake regulator protein, Fur, controls iron metabolism (49). Fur activity depends on cellular free iron, which acts as a corepressor of transcription (40). Fur also activates gene expression via RNA polymerase recruitment, blocking repressor access, or transcriptional repression of RyhB sRNA (36). RyhB base pairing stimulates the degradation of mRNAs encoding nonessential iron-dependent proteins, increasing iron availability for essential processes (50). The goal of the present study was to explore the effects of CsrA on E. coli iron homeostasis. Unlike Fur and RyhB, we show that CsrA is not responsive to iron availability but nevertheless exerts biologically important effects on iron homeostasis and growth by regulating the expression of ISPs.

## RESULTS

### CsrA regulates expression of iron storage genes independently of Fur.

Recent transcriptomics and other studies identified several potential mRNA targets of CsrA-mediated regulation that are involved in iron uptake and storage, suggestive of a regulatory role in iron metabolism (4, 19, 51). To further investigate this possibility, twelve of these genes were chosen for expression analysis (see Table S1 in the supplemental material). Altogether, eleven translational ′*lacZ* fusions and one C-terminal 3×FLAG-tagged reporter fusion were constructed and integrated in single copy into the E. coli chromosome.

10.1128/mBio.01034-19.1 TABLE S1 Historical evidence of CsrA effects on genes related to iron metabolism. Download Table S1, DOCX file, 0.1 MB. Copyright © 2019 Pourciau et al. 2019 Pourciau et al. This content is distributed under the terms of the Creative Commons Attribution 4.0 International license.

We assessed the expression of the following genes, which encode the indicated proteins. We assessed four iron transport genes: (i) *fhuE*, an outer membrane receptor for the xenosiderophores coprogen, ferrioxamine B, and rhodotorulic acid; (ii) *fecB*, the periplasmic ferric-citrate transporter; (iii) *fepA*, the ferrienterobactin outer membrane receptor; and (iv) *fhuA*, the outer membrane transporter for ferrichrome. Four genes encoding ISPs were assessed: (i) *ftnA*, the primary reservoir for iron in E. coli K-12; (ii) *ftnB*, a ferritin-like ISP; (iii) *bfr*, a heme-sequestering bacterioferritin; and (iv) *dps*, an iron-sequestering and nonspecific DNA-binding protein that protects DNA in starved cells. Two genes related to siderophores were investigated: (i) *fes*, enterobactin esterase, which hydrolyzes the siderophore backbone to release bound iron; and (ii) *entC*, an isochorismate synthase involved in enterobactin biosynthesis. Lastly, the genes encoding Fur and an Fe-S cluster assembly protein, SufA, were examined.

**FIG 1:**
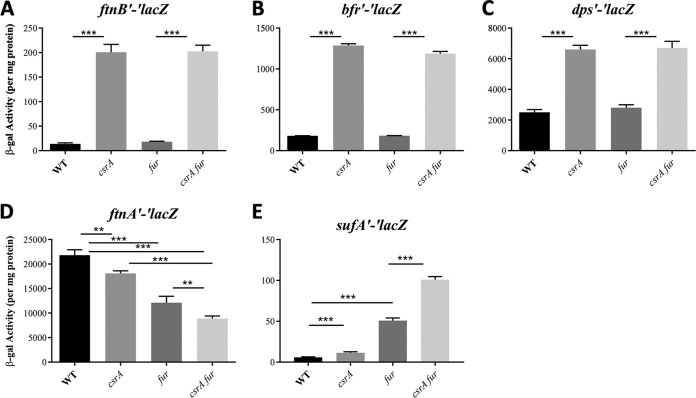
(A to E) Effects of *csrA* and *fur* mutations on expression of translational *lacZ* fusions. Mean β-galactosidase activities ± standard deviation were determined from exponential-phase cultures (OD_600_ of 0.5) grown in LB. Each bar shows the mean and standard deviation from four separate experiments. Statistical significance was determined using unpaired *t* tests and denoted as follows: ***, *P* < 0.001; **, *P* < 0.002.

Expression of the reporter fusions was monitored in the wild-type (WT) strain background MG1655 and in isogenic *csrA*, *fur*, and *csrA fur* mutant strains. These studies confirmed CsrA-dependent regulation of five iron metabolism genes (Fig. 1A to E). Translational fusions for three iron storage genes (*ftnB*, *bfr*, and *dps*) were strongly repressed by CsrA (Fig. 1A to C). The *ftnB*′*-*′*lacZ* fusion exhibited the most striking effect, with a 14-fold increase in the *csrA* mutant. Fur did not affect *ftnB*′-′*lacZ* expression under the conditions of this study (Fig. 1A). Expression of *bfr*′-′*lacZ* increased 7-fold in the *csrA* mutant (Fig. 1B). The *bfr* gene has been reported to be posttranscriptionally regulated by the Hfq-dependent sRNA RyhB, which is repressed by Fur (52). In contrast, we did not observe Fur-dependent regulation of the *bfr*′-′*lacZ* fusion. As the RyhB binding site in *bfr* mRNA has yet to be identified, it is conceivable that our fusion did not encompass that region. Finally, an ∼3-fold increase in the *csrA* mutant was observed for *dps*′-′*lacZ*, which did not respond to Fur (Fig. 1C).

Moderate CsrA-dependent effects were observed for *ftnA* and *sufA* fusions (Fig. 1D and E). The *ftnA*′-′*lacZ* showed positive effects of CsrA on its expression, which were retained in the Δ*fur* mutant (Fig. 1D). Thus, CsrA has a minor role in activating *ftnA* gene expression that is mediated independently of Fur. The *sufA*′-′*lacZ* fusion exhibited modest repression via CsrA in the *fur* WT and mutant backgrounds (Fig. 1E). The *entC*, *fhuE*, *fepA*, *fes*, and *fhuA* translational fusions were all repressed by Fur but were not regulated by CsrA (Fig. S1A to E). Expression of *fur*′*-*′*lacZ* was unaffected by CsrA (Fig. S1F). Altogether, these findings demonstrate that CsrA effects on iron-related gene expression are mediated independently of Fur.

10.1128/mBio.01034-19.4 FIG S1 Effects of *csrA* and *fur* mutations on expression of translational *lacZ* fusions (A to F) and C-terminal-tagged FecB-3×FLAG (G). β-Galactosidase activities ± standard deviations were determined in exponential-phase cultures (OD_600_ of 0.5). Each bar shows the mean and standard deviation from four separate experiments. Statistical significance was determined using unpaired *t* tests and is denoted as follows: ***, *P* < 0.001. (G) FecB levels from exponentially growing cultures supplemented with 1 mM sodium citrate to induce expression were normalized to the RpoB signal. This experiment was repeated twice with essentially identical results. Mean fold change is indicated with respect to WT levels. Download FIG S1, TIF file, 2.9 MB. Copyright © 2019 Pourciau et al. 2019 Pourciau et al. This content is distributed under the terms of the Creative Commons Attribution 4.0 International license.

### Growth phase-dependent regulation of *ftnB*, *bfr*, and *dps* expression by CsrA.

**FIG 2:**
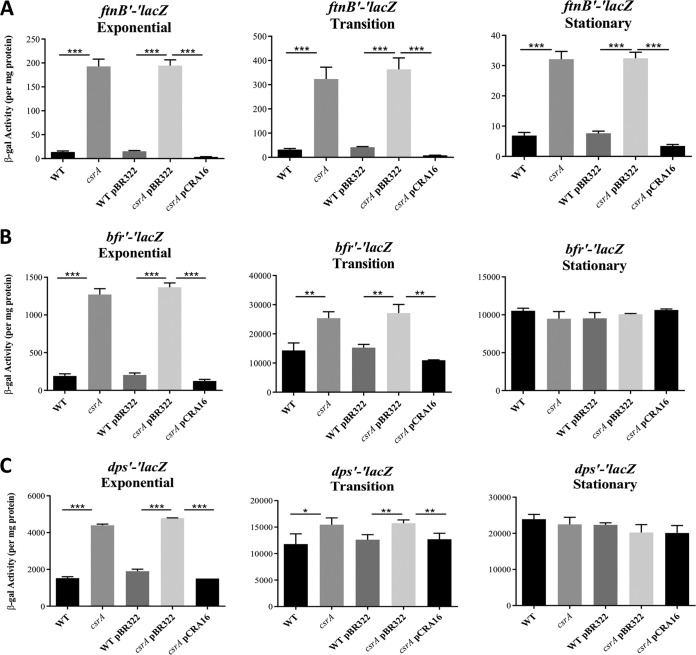
Effects of *csrA* mutation, vector control (pBR322), and *csrA* complementation (pCR16) on expression of the *ftnB* (A), *bfr* (B), and *dps* (C) translational *lacZ* fusions. β-Galactosidase activities ± standard deviations were determined in exponential-phase (OD_600_ of 0.5), transition to stationary-phase (OD_600_ of 1.5), and overnight cultures grown in LB. Each bar shows the mean and standard deviation from four separate experiments. Statistical significance was determined using unpaired *t* tests and denoted as follows: ***, *P* < 0.001; **, *P* < 0.002; *, *P* < 0.05.

Having identified three iron storage genes, *ftnB*, *bfr* and *dps*, that are repressed by CsrA, we assessed their expression during exponential phase, the transition from exponential to stationary phase, and stationary-phase growth at 24 h. Complementation tests were performed using plasmid-borne *csrA* (pCRA16) versus the empty vector (pBR322). The *ftnB*, *dps*, and *bfr* fusions all exhibited growth phase-dependent regulation by CsrA, which was abolished by complementation (Fig. 2). CsrA repressed the three genes maximally during exponential growth, with weaker effects in the transition to stationary phase and in stationary phase. The *bfr* and *dps* fusions were not regulated by CsrA during stationary phase (Fig. 2B and C), while *ftnB* repression decreased from 14-fold in the exponential phase to ∼4-fold at 24 h (Fig. 2A). *ftnB* expression peaked during the transition to stationary phase and remained higher in the *csrA* mutant in all stages of growth (Fig. 2A). *bfr* expression levels were lowest in exponential phase, higher at transition to stationary phase, and then decreased modestly in stationary phase (Fig. 2B). Finally, the growth-phase expression pattern of *dps* reflected what was already known; Dps levels are low during exponential growth and increase upon entry to stationary phase (Fig. 2C) (53). Thus, CsrA plays a particularly important role in limiting the expression of these three genes during exponential growth.

### CsrA binds with high affinity and specificity to 5′ UTRs of *ftnB*, *dps*, and *bfr* transcripts.

**FIG 3:**
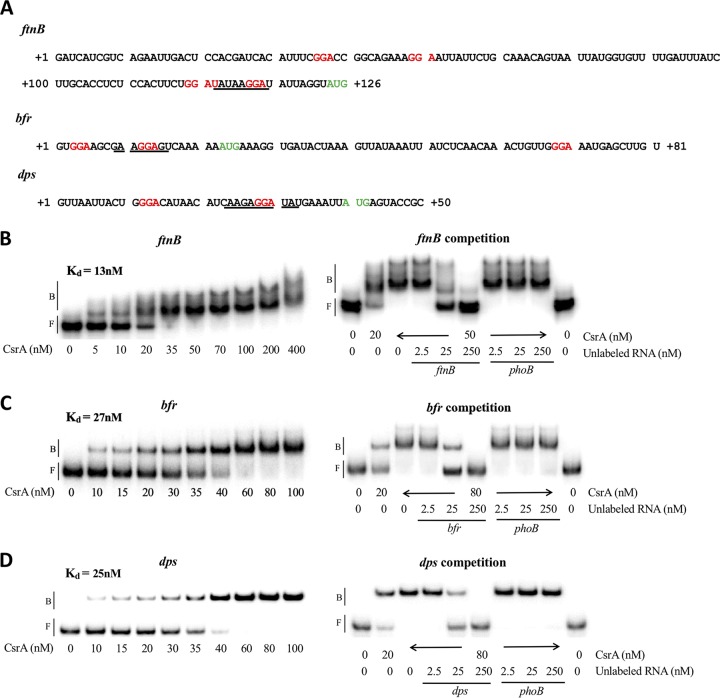
CsrA binding and competition reactions with *ftnB* (B), *bfr* (C), and *dps* (D) transcripts. (A) EMSA probe sequences with GGA sequences that are potential CsrA binding sites shown in red, translation initiation sites in green, and Shine-Dalgarno (SD) sequences underlined. (B to D) 5′-End-labeled transcripts (0.5 nM) were incubated with CsrA at the concentrations shown. Competition reactions were performed in the presence of specific or nonspecific (*phoB*) unlabeled competitor RNAs at the concentrations shown. The positions of free (F) and bound (B) RNA are marked with vertical bars.

Although a CLIP-seq assay did not identify *in vivo* CsrA binding, previous transcriptomics data demonstrated that CsrA affects the stability and abundance of *ftnB* and *dps* mRNA (19), suggestive of posttranscriptional regulation. To explore the possible binding of CsrA to *ftnB*, *bfr*, and *dps* transcripts, electrophoretic gel mobility shift assays (EMSA) were performed. CsrA exhibited high-affinity binding to all three RNAs (Fig. 3B to D). A nonlinear least-squares analysis of the data yielded apparent *K_D_* (equilibrium binding constant) values of 13 nM, 27 nM, and 25 nM for the *ftnB*, *bfr*, and *dps* RNAs, respectively, similar to those for known CsrA mRNA targets (26, 54). The *bfr* and *dps* mRNAs displayed a single shift as CsrA concentration was raised to 100 nM, while two distinct shifted species and a faint minor form were observed for *ftnB* at 50 nM and higher, suggesting that more than one CsrA dimer may bind to this RNA. Examination of the *ftnB* sequence identified four potential CsrA binding sites, while the *bfr* and *dps* sequences contained three and two potential CsrA binding sites, respectively (Fig. 3A). Competition assays performed with specific (self) and nonspecific (*phoB*) unlabeled competitor transcripts confirmed that binding is specific in all cases (Fig. 3B to D).

Taken together with reporter fusion data (Fig. 1 and 2) and transcriptomics studies (19), the high-affinity binding of CsrA to *ftnB* mRNA suggests that CsrA may directly regulate expression of this gene by binding to its 5′ UTR. CsrA also bound tightly to the 5′ UTR of *dps* mRNA, although the interpretation of this interaction is less clear. While CsrA negatively affects *dps* RNA abundance and translation (as measured by changes in ribosome occupancy), it increases *dps* mRNA stability (19). It is likely that CsrA binding to the *dps* 5′ UTR is responsible for one or more of these effects. Finally, the high-affinity binding of CsrA to the 5′ UTR of the *bfr* transcript, along with increased mRNA abundance in the *csrA* mutant (19), suggests that CsrA may directly repress *bfr* expression by binding its 5′ UTR.

### CsrA directly represses translation of *ftnB*, *dps*, and *bfr* via their 5′ UTRs.

**FIG 4:**
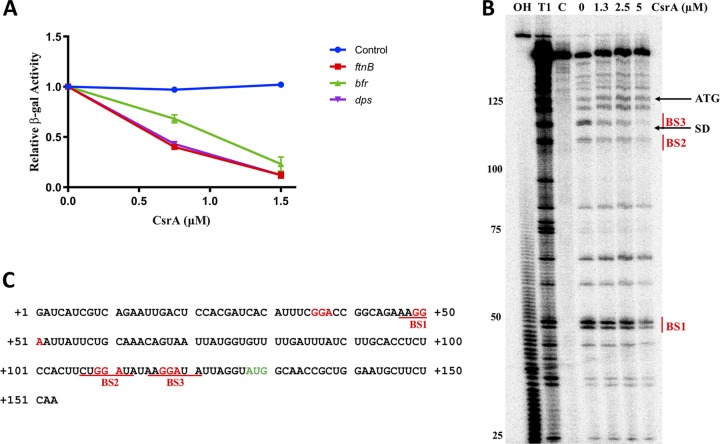
Repression of *ftnB*, *dps*, and *bfr* translation *in vitro* by CsrA and RNase T1 footprinting of *ftnB* RNA. (A) CsrA effects on cell-free protein synthesis of β-galactosidase from plasmid templates containing ′*lacZ* fused to the 5′ UTR of each mRNA target and transcribed from a T7 promoter. Relative β-galactosidase activity depicts the mean and standard deviation of activity relative to reaction mixtures lacking CsrA. (B) CsrA-*ftnB* RNA footprint. 5′-End-labeled *ftnB* RNA was treated with RNase T1 ± CsrA, as shown. Partial alkaline hydrolysis (OH) and RNase T1 digestion (T1) ladders, as well as a control lane without treatment (C), are shown. Positions of the *ftnB* start codon (ATG) and the Shine-Dalgarno (SD) sequence are marked. Residues protected from RNase T1 cleavage by CsrA are indicative of binding at three sites, BS1 to -3, and are shown. Numbering is with respect to the start of *ftnB* transcription. (C) Sequence of *ftnB* leader RNA. Position of the translation initiation codon indicated in green, GGA sequences are shown in red, and the deduced CsrA binding sites are indicated.

To assess whether the regulatory effects of CsrA on *ftnB*, *bfr*, and *dps* are mediated posttranscriptionally, without the requirement for an intermediate regulatory gene, we used the PURExpress system to measure *lacZ* expression from reporter fusions carried on plasmid templates. Reporter sequences contained a T7 promoter fused to the 5′ UTR of each gene and a few codons of the coding region (Fig. 3A). In all cases, with the exception of the control (*pnp*′*-*′*lacZ*), expression was inhibited by CsrA. Thus, CsrA represses translation of *ftnB*, *bfr*, and *dps* via their 5′ UTRs (Fig. 4A). The *in vitro* translational repression exhibited by the *dps* reporter was similar to that by the *ftnB* reporter. This pattern of regulation differs from what was seen in the *in vivo* translational fusion assays, where *dps* demonstrated substantially less CsrA-dependent repression than *ftnB* (Fig. 2). This difference may result from increased *dps* mRNA stability, which was observed previously in the *csrA* mutant strain (19), or it might involve indirect effects of CsrA on *dps* transcription *in vivo*.

To identify the precise CsrA interaction site(s) for the most strongly regulated gene, *ftnB*, RNA footprinting experiments were performed. Three regions were protected from RNase T1‐mediated cleavage with increasing concentrations of CsrA (Fig. 4B). The protected nucleotides correspond to three of the four GGA motifs, indicating that CsrA has three authentic binding sites in the *ftnB* 5′ UTR (Fig. 4C) (BS1, BS2, and BS3). BS3, which overlaps the *ftnB* Shine-Dalgarno (SD) sequence, exhibited the strongest protection by CsrA. This finding provides a molecular mechanism for *ftnB* translational repression by CsrA, observed *in vitro* (Fig. 4A). The multiple protected regions are consistent with EMSA results, which show that more than one complex is formed between CsrA and the *ftnB* 5′ UTR (Fig. 3B).

### Overexpression of iron storage genes in the *csrA* mutant increases cellular iron levels.

**FIG 5:**
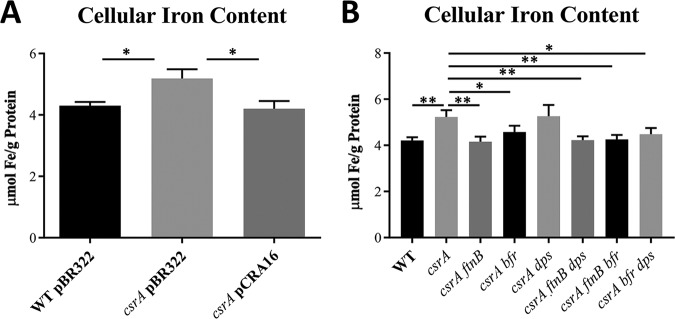
Cellular iron content in E. coli strains. Cells were harvested during exponential growth (OD_600_ of 0.5) in LB, lysed with 30% nitric acid, and iron was quantified via ICP-OES. (A) Comparison of iron levels in WT, *csrA* mutant, and *csrA*-complemented strains. (B) Iron content of WT, *csrA*, and combinatorial mutants in genes for iron storage proteins. Each bar shows the mean and standard deviation from two separate biological experiments with paired technical replicates. Statistical significance was determined using unpaired *t* tests and is denoted as follows: **, *P* < 0.002; *, *P* < 0.05.

To determine the effect of CsrA regulation of iron storage genes on cellular iron levels, exponential-phase cultures were analyzed for iron content by inductively coupled plasma optical emission spectrometry (ICP-OES). Cellular iron content was ∼25% higher in the *csrA* mutant (5.2 ± 0.15 μmol Fe/g protein) than in the WT (4.2 ± 0.07 μmol Fe/g protein) and was restored to the WT level upon complementation (Fig. 5A). This result is consistent with the increased expression of *ftnB*, *bfr*, and *dps* in the *csrA* mutant, which should increase its capacity for iron storage. To test this hypothesis, deletions of the chromosomal *ftnB*, *bfr*, and *dps* genes were introduced, and cellular iron levels were measured. Iron levels in the *csrA* mutant approached WT levels when FtnB and/or Bfr production was abolished (Fig. 5B), suggesting that increased iron accumulation in the *csrA* mutant is caused by the overproduction of these ISPs. The *dps* mutation had no substantial effect on iron levels, alone or in combination with *ftnB* or *bfr*. Notably, the increased iron in the *csrA* mutant may not be biologically available. In fact, excessive iron sequestration by FtnB and Bfr in the *csrA* mutant might inhibit iron-dependent cellular processes, to the detriment of cell growth.

### Growth under iron-limiting conditions is compromised by *csrA* mutation and restored by deletion of *ftnB* and *bfr*.

To test the above hypothesis, we monitored the growth kinetics of WT, *csrA*, *ftnB*, *bfr*, and/or *dps* mutants under iron-replete and iron-limiting conditions in both rich and minimal media. Growth in rich medium was assessed by tracking total cell protein of cultures grown in LB (for iron-replete conditions) and LB containing 400 μM 2,2′-dipyridyl (DIP) (for iron-limiting conditions). Growth in minimal medium was evaluated by measuring the optical density at 600 nm (OD_600_) in MOPS (morpholinepropanesulfonic acid) medium supplemented with 100 μM FeSO_4_ (iron replete) or 0.05 μM FeSO_4_ (iron limiting) and 0.2% glucose (55). However, biofilm formation compromised growth measurements (*A*_600_) in MOPS medium. Under this condition, biofilm was apparently dependent upon the formation of curli fimbriae and was abolished by deletion of *csgA* (data not shown). Thus, all strains examined in MOPS medium were constructed in a *csgA* background.

**FIG 6:**
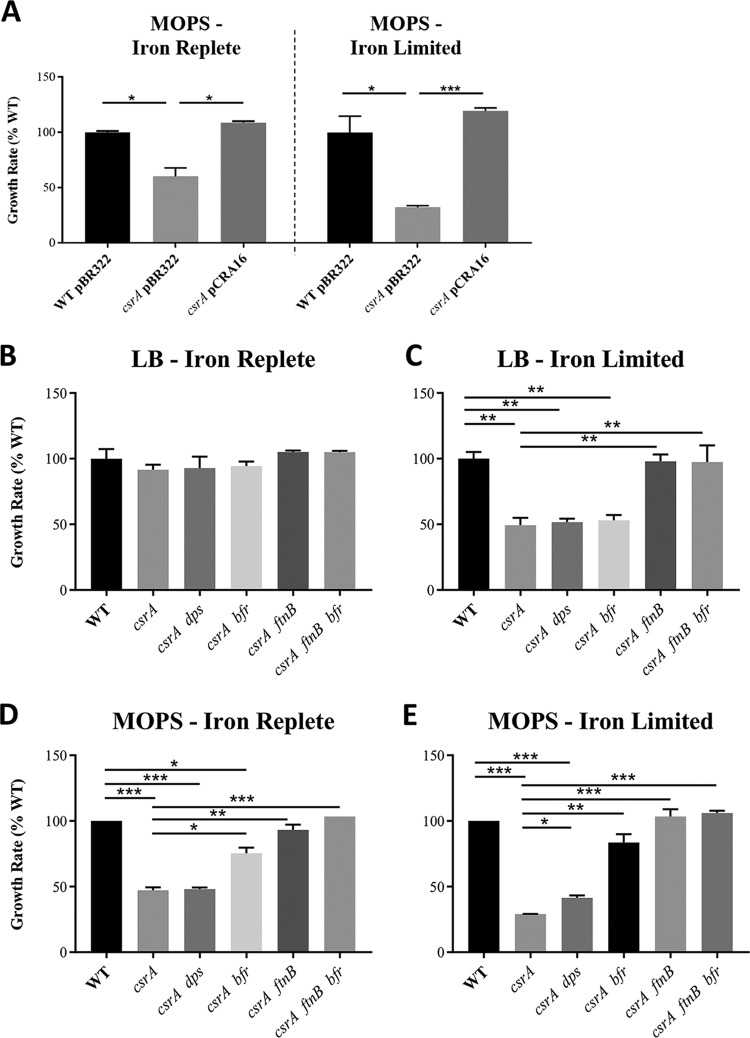
Growth rates (μ) of exponential-phase E. coli strains in LB ± 400 μM DIP and MOPS minimal medium with replete (100 μM) or limiting (0.05 μM) FeSO_4_ as indicated. (A) WT, *csrA* mutant, and *csrA*-complemented strains in MOPS. (B and C) WT, *csrA* mutant, and *ftnB*/*dps*/*bfr* combinatorial mutants in LB. (D and E) WT, *csrA* mutant, and *ftnB*/*dps*/*bfr* combinatorial mutants in MOPS. Each bar represents the mean and standard deviation from 4 separate experiments. See Fig. S2 in the supplemental material for associated growth curves. Statistical significance was determined using unpaired *t* tests. ***, P < 0.001; **, *P* < 0.002; *, *P* < 0.05.

Growth of the WT and *csrA* strains was inhibited under iron-limiting conditions in both media, and these growth defects were particularly severe in the *csrA* mutant (Fig. 6; Fig. S2). To assess the contribution of each iron storage gene to the growth inhibition exhibited by the *csrA* mutant, deletions in the chromosomal *ftnB*, *bfr*, and *dps* genes were introduced into the WT and *csrA* mutant. Each strain was grown under iron-replete and iron-limiting conditions in both the rich (Fig. 6B and C; Fig. S2B and C) and minimal (Fig. 6D and E; Fig. S2D and E) media. The inactivation of *ftnB*, *bfr*, or *dps* had no significant effect on growth of the WT strain under any condition (Fig. S2B to D). However, introduction of these knockouts into the *csrA* mutant led to partial or complete restoration of the WT growth rate in MOPS medium with limiting iron (Fig. 6E). In rich medium, the *csrA* mutant exhibited reduced growth only when iron was limiting, and this growth defect was completely recovered upon inactivation of *ftnB* alone; *dps* or *bfr* alone did not restore growth (Fig. 6B and C). Interestingly, the *csrA* mutant displayed a growth defect in the minimal medium under both iron conditions, although the effects were more pronounced in the iron-limited MOPS (Fig. 6D and E). Inactivation of *ftnB* in the *csrA* mutant recovered the growth rate completely in both iron-limited and iron-replete minimal media. The introduction of a *bfr* mutation to the *csrA* mutant partially restored growth in iron-replete and iron-limited MOPS (Fig. 6D and E). The *dps* mutation only improved growth of the *csrA* mutant in MOPS medium with limiting iron, and this effect was weak (Fig. 6D and E).

10.1128/mBio.01034-19.5 FIG S2 Growth curves of E. coli strains in LB ± 400 μM DIP and MOPS minimal medium with replete or limiting FeSO_4_ as indicated. (A) WT, *csrA* mutant, and *csrA*-complemented strains in MOPS. (B, C) WT, *csrA* mutant, and *ftnB*/*dps*/*bfr* combinatorial mutants in LB. (D and E) WT, *csrA* mutant, and *ftnB*/*dps*/*bfr* combinatorial mutants in MOPS. Each line represents the mean and standard deviation from 4 separate experiments. Download FIG S2, TIF file, 2.9 MB. Copyright © 2019 Pourciau et al. 2019 Pourciau et al. This content is distributed under the terms of the Creative Commons Attribution 4.0 International license.

### Iron availability has negligible effects on CsrA/B/C levels.

**FIG 7:**
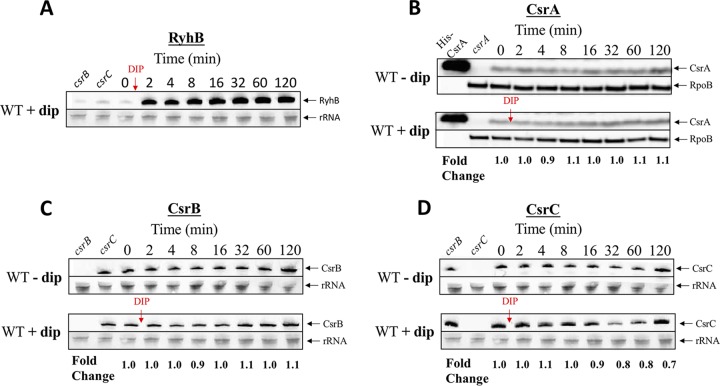
Effects of the addition of 250 μM 2,2′‐dipyridyl (DIP) on RyhB (A), CsrA (B), CsrB (C), and CsrC (D) levels at mid-exponential-phase growth (OD_600_ of 0.5). CsrA protein was detected by Western blotting and RyhB and CsrB/C sRNAs were detected by Northern blotting. Values were normalized against RpoB or rRNA, respectively, to calculate specific levels of CsrA, CsrB, and CsrC. Fold change shows the effects of DIP on specific CsrA, CsrB, and CsrC levels versus controls lacking DIP. Each experiment was repeated twice with essentially identical results. (A) RyhB sRNA response to DIP as a positive control for iron sequestration. (B) Purified C-terminally His_6_-tagged CsrA protein served as a marker for the Western blotting. (A, C, and D) RNA isolated from strains deleted for *csrB* and for *csrC* is shown in the first and second lanes, respectively.

A fundamental feature of Csr systems is their ability to integrate a variety of nutritional cues and signals and to interact with other regulators in diverse circuitry (10, 16, 29). Therefore, we sought to determine whether iron availability affects levels of Csr system components. Identically growing exponential-phase cultures of WT E. coli were treated with or without DIP (250 μM), and CsrA/B/C levels were measured. RyhB levels served as a positive control for the iron starvation response (Fig. 7A) (52). CsrA was determined by Western blotting using polyclonal anti-CsrA antibodies and normalized to RpoB (Fig. 7B). CsrA levels were unaffected by DIP addition, indicating that CsrA expression is not regulated in response to a change in iron availability. Northern blotting (Fig. 7C and D) showed that CsrB levels were unaffected by iron availability, although CsrC levels dropped slightly ∼30 min after the addition of DIP. CsrB/C sRNAs function via CsrA, and CsrB is a more effective antagonist of CsrA than CsrC (56). Thus, the minor change in CsrC levels at 30 min might not have a significant impact on cellular physiology. In summary, it appears that iron availability has negligible effects on the key components of the Csr system and is not one of the cues to which the Csr system responds.

### CsrA regulates resistance of E. coli against killing by H_2_O_2_.

**FIG 8:**
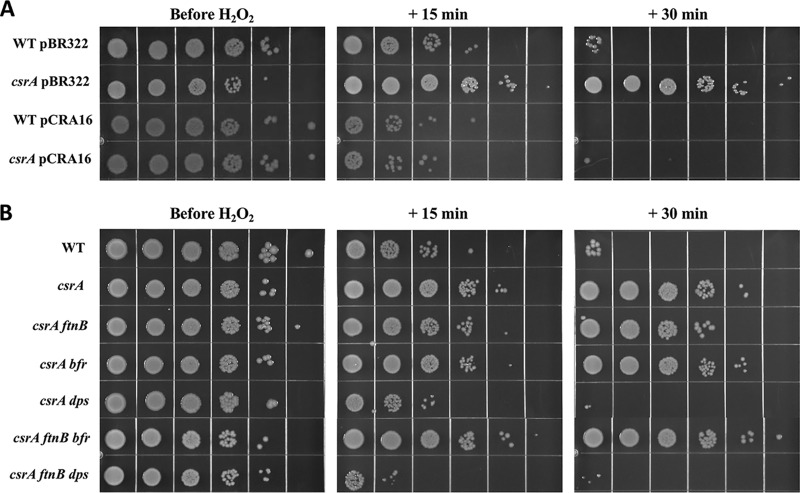
Survival of WT (MG1655) and *csrA* mutant strains upon exposure to H_2_O_2_. (A) WT and *csrA* mutant strains carrying pBR322 or plasmid pCRA16 (*csrA*^+^). (B) WT, *csrA*, and *ftnB*, *dps*, and *bfr* combinatorial mutants. Strains were grown in LB to mid-exponential phase (OD_600_ of 0.5) and exposed to 12.5 mM H_2_O_2_. Samples were collected 0, 15, and 30 min after addition of H_2_O_2_, washed, and 10-fold serially diluted. The 10^−1^ to 10^−6^ dilutions were plated and grown for 18 h on LB agar at 37°C.

The finding that CsrA represses *sufA* (Fig. 1E), which encodes a protein involved in Fe-S assembly under oxidative stress (43), suggested that CsrA may play a role in regulating resistance against oxidative damage. To test this hypothesis, we exposed several isogenic strains to H_2_O_2_ during the exponential phase of growth and monitored its effect on viability. As suspected, the *csrA* mutant was considerably more resistant to oxidative stress than the WT strain (Fig. 8). Complementation restored sensitivity of the *csrA* mutant to H_2_O_2_, confirming a role for CsrA in repressing oxidative stress resistance. While deleting *ftnB* and/or *bfr* did not restore H_2_O_2_ sensitivity to the *csrA* mutant, the loss of *dps* caused a marked increase in H_2_O_2_ susceptibility, resulting in greater sensitivity even than in the *csrA* WT strain (Fig. 8B). Perhaps this is not surprising, given the importance of Dps in protection against oxidative stress (57). Finally, the WT and *csrA* mutant strains were tested for survival against H_2_O_2_ during early stationary phase. Unexpectedly, the *csrA* mutant was considerably more sensitive than WT to H_2_O_2_ at this stage of growth (see Fig. S3), opposite of its response in the exponential phase (Fig. 8). These observations are consistent with recent findings from Salmonella enterica that CsrA is a flexible regulator whose regulon varies under different growth or physiological conditions (58).

10.1128/mBio.01034-19.6 FIG S3 Survival of WT (MG1655) and *csrA* mutant strains upon exposure to H_2_O_2_. Strains were grown in LB to early stationary phase (OD_600_ of 2.5) and exposed to 50.0 mM H_2_O_2_. Samples were collected 0, 15, and 30 min after addition of H_2_O_2_, washed, and 10-fold serially diluted. The 10^−2^ to 10^−7^ dilutions were plated and grown for 18 h on LB agar at 37°C. Download FIG S3, TIF file, 2.2 MB. Copyright © 2019 Pourciau et al. 2019 Pourciau et al. This content is distributed under the terms of the Creative Commons Attribution 4.0 International license.

## DISCUSSION

Here, we show that CsrA regulates at least 5 genes that participate in iron storage and utilization (Fig. 1). Repression of *ftnB* and *bfr* by CsrA affects cellular iron content (Fig. 5) and is required for optimal growth under iron limitation and in minimal medium (Fig. 6). In other words, repression of *ftnB* and *bfr* by CsrA positions the Csr system to guide intracellular iron flux toward growth-promoting processes. CsrA is known to broadly repress gene expression involved in stress responses and in the stationary phase of growth. Furthermore, the Csr system itself does not appear to respond to iron availability (Fig. 7). In contrast, CsrB and CsrC sRNAs are known to be expressed under conditions of stress or nutrient limitation, and by sequestering CsrA, these sRNAs diminish CsrA-mediated regulation of gene expression when growth is no longer a priority (4, 5, 30). This regulatory arrangement no doubt allows the cellular capacity for iron storage to be governed in part by nutritional and growth phase conditions that influence the Csr system (29).

To our knowledge, the present studies represent the first evidence that the repression of gene expression by CsrA can be necessary for robust exponential growth, although modest growth support via CsrA-mediated repression has been observed (59). While the *csrA*::*gm* allele used in this study results in a much less severe growth defect than seen in a Δ*csrA* strain, even when the *csrA*::*gm* strain was grown under limiting iron conditions, we posit that the findings obtained with the *csrA*::*gm* mutant are nevertheless important for the following reasons. (i) Most likely, there is no environmental condition under which CsrA activity is absent, but there may be conditions under which reduced CsrA activity impacts iron requirements. (ii) In considering the development of CsrA inhibitors (60), complete inhibition of CsrA activity may not be required to achieve useful therapeutic effects, because human and animal body compartments tend to contain little available iron (see related discussion below).

The high-affinity binding of CsrA to *ftnB*, *dps*, and *bfr* transcripts (Fig. 3), taken together with *in vitro* translation results (Fig. 4A), indicates that CsrA represses translation of *ftnB*, *bfr*, and *dps* mRNAs by binding to the 5′ UTR of each transcript. Furthermore, RNase T1 footprinting revealed that CsrA binds to the *ftnB* SD sequence (Fig. 4B), which should directly impede ribosome loading. Translational repression of *hfq*, *glgC*, *cstA*, and *pgaA* expression is similarly mediated by CsrA binding to sites overlapping the SD sequences (21, 54, 61). Both *bfr* and *dps* transcripts contain potential CsrA binding sites overlapping their SD sequences (Fig. 3A), which might mediate translational repression by a similar mechanism (Fig. 4C).

The overexpression of *ftnB* and *bfr* in the *csrA* mutant caused excessive accumulation of cellular iron (Fig. 5). The *csrA* mutation also inhibited growth under iron-limiting conditions, apparently by altering iron bioavailability (Fig. 6). The relative magnitude of CsrA-dependent repression of genes for ISPs (*ftnB* > *bfr* > *dps*) mirrored the effects of these genes on cellular iron content and growth (*ftnB* > *bfr* > *dps*) (Fig. 1A to C, 5B, and 6C to E). We propose that during exponential growth, repression of the ISPs, particularly FtnB and Bfr, prevents the sequestration of iron away from iron-dependent growth processes. Importantly, the regulatory effects of CsrA on these genes became weaker or were eliminated as cultures entered the stationary phase of growth (Fig. 2), during which time E. coli becomes resistant to a variety of stressors (62).

The complex and dissimilar regulation of the E. coli ISPs seemingly complicates the interpretation of their individual physiological roles (34). *ftnA* expression is primarily regulated by Fur-mediated transcriptional activation, coupling FtnA levels to iron availability (63). At high iron levels, FtnA serves as the primary reservoir in E. coli, storing up to 50% of cellular iron (64). Under low to moderate iron levels, Bfr can become the principal ISP (65). Notably, *bfr* expression is induced by stresses, including osmotic and heat stress, while *ftnA* expression is not (66). Likewise, *ftnB* transcription can be driven by a σ^E^-dependent promoter (67), linking FtnB production to extracytoplasmic stress, or from a σ^D^-dependent promoter, as part of the Cpx regulon, connecting it to cell envelope damage (68). *dps* transcription is induced under oxidative stress by the transcriptional activator OxyR (69) and by σ^S^ during the stationary phase of growth (70). This regulatory diversity implies that individual ferritins are beneficial under different physiological conditions.

The strongest regulation by CsrA observed here was that of *ftnB* (Fig. 1A). FtnB is the major Fe^2+^ donor for the repair of oxidatively damaged Fe-sulfur clusters in *Salmonella*, although this is yet to be confirmed in E. coli (71). These findings raise the possibility that a primary role of CsrA in iron homeostasis is related to oxidative stress and repair response. CsrA also repressed *bfr* and *dps* (Fig. 1B and C), whose protein products appear to play modest roles in iron storage in E. coli and are linked to stress responses (64). CsrA repressed expression of *sufA* (Fig. 1E) and negatively affected the levels of *sufABCDE* operon transcripts (19), which encode proteins responsible for Fe-S cluster assembly under stress conditions. Recent research suggests that FtnB, Bfr, and Dps may each function to donate iron to the Suf Fe-S cluster biogenesis pathway (72). Thus, the present findings are consistent with a regulatory role of CsrA in the prevention and repair of oxidative damage.

**FIG 9:**
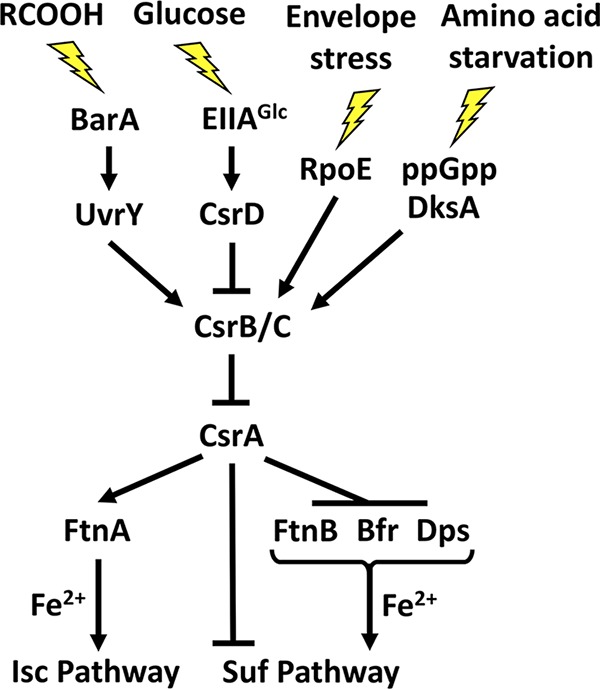
A model for regulation of iron homeostasis by CsrA in E. coli. During exponential growth and in the absence of stress, CsrA represses *ftnB*, *bfr*, *dps*, and *sufA*, inhibiting the delivery of iron to and the expression of the stress-resistant Suf Fe-S cluster assembly pathway. CsrA also activates the expression of *ftnA*, which donates iron to the Isc pathway. Upon stress or stationary-phase growth, CsrB/C sRNAs accumulate and sequester CsrA, which derepresses translation of *ftnB*, *bfr*, and *dps*, helping to divert iron from the housekeeping Isc Fe-S cluster assembly pathway and toward the Suf pathway. Activation of gene expression and iron transfer are indicated by arrows; repression is shown by a T bar.

CsrA modestly activated *ftnA* expression, in contrast to its stronger negative effects on the genes for the other ISPs. Unlike FtnB, Bfr, and Dps, FtnA does not appear to donate iron to the Suf Fe-S cluster biogenesis pathway (72). In fact, FtnA facilitates the assembly of iron-sulfur clusters via the Isc pathway under normal physiological conditions (73). Although Suf Fe-S biogenesis is preferred to Isc assembly during stress, the Suf pathway fails to fully mature some Fe-S proteins and may only meet the minimal Fe-S requirements for growth (43, 48, 74). As such, the activation of FtnA and repression of FtnB, Bfr, Dps, and SufA by CsrA may assist in directing iron stores toward the Fe-S assembly pathway that is most beneficial for exponential growth (Fig. 9). These findings suggest an explanation for the *csrA* growth defect in minimal medium, even when iron is replete. The loss of CsrA activation of FtnA and repression of FtnB, Bfr, Dps, and SufA apparently result in an inefficient utilization of available iron, which is particularly detrimental in minimal medium, where the demand for Fe-S proteins involved in biosynthetic processes may be high (75).

Interestingly, the ferritins are not the first example of CsrA mediating opposing regulatory effects on proteins having identical or highly related activities. For example, expression of the major phosphofructokinase, PfkA, is activated by CsrA, while the minor and σ^s^-inducible enzyme, PfkB, is repressed by CsrA (1, 19, 76). The different allosteric responses of these enzymes to key metabolites are most likely the basis of their distinct regulatory patterns. To our knowledge, in such cases, CsrA consistently serves to repress the expression of genes associated with stationary-phase growth and/or stress responses, while tending to activate genes that are needed to support exponential growth.

The dramatically increased resistance of the *csrA* mutant to H_2_O_2_ during exponential growth demonstrates a regulatory role of CsrA in the resistance of E. coli to oxidative damage (Fig. 8). This is further supported by transcriptomics studies, which found increased steady-state mRNA levels for genes involved in antioxidant defense in the *csrA* mutant during exponential-phase growth, including the transcriptional activator of the superoxide response regulon, *soxS*, superoxide dismutases *sodA* and *sodC*, and catalase *katE* (19). CsrA also copurifies with mRNAs encoding the superoxide dismutase SodB and the catalase-peroxidase KatG (4). A recent study also provided evidence for a role for CsrA in repressing the oxidative stress response in *Salmonella* (58). The repression of many genes involved in oxidative stress response pathways by CsrA suggests that ungoverned expression of these processes may be detrimental during exponential growth and in the absence of stress, when conditions favor high CsrA activity (30). Finally, the unexpected finding that the *csrA* mutant became less resistant than the WT to H_2_O_2_ in the stationary phase of growth (see Fig. S3 in the supplemental material) suggests that CsrA also performs important yet unexplained functions required for oxidative stress resistance in the stationary phase.

Commensal E. coli is ubiquitous in the gastrointestinal tracts of mammals and seemingly has colonized every mammalian species on the planet (77). While much research has been devoted to the role of CsrA in regulating virulence gene expression in pathogenic *Gammaproteobacteria* (10), little is known about its role in commensal colonization. E. coli inhabits the large intestine, which is primarily anoxic and nutrient limited. In this environment, soluble free iron is a contested and often limited resource (78). In addition, reactive oxygen species are scarce in the healthy intestinal lumen, although this can change during inflammation. For commensal microbes, iron is critical for enzyme function and energy generation. By repressing genes with the potential to sequester iron or divert iron to unnecessary stress response processes, CsrA fosters E. coli growth under iron-limiting laboratory conditions. We propose that it may play a similar role during colonization and survival in the large intestine.

## MATERIALS AND METHODS

### Bacterial strains and culture conditions.

The bacterial strains and plasmids used in this study are listed in Table S2 in the supplemental material. All media and medium components were prepared using Nanopure water, 18.2 MΩ/cm (Barnstead). Bacterial strains were grown in LB broth (5 g yeast extract, 10 g Bacto tryptone, and 10 g NaCl per liter double-distilled water [ddH_2_O]), pH 7.4, at 37°C, with shaking (250 rpm), unless otherwise indicated. For growth curves under iron-limiting and iron-replete conditions, MOPS defined medium (79) with 0.2% glucose was used; FeSO_4_ was supplemented at the concentrations as indicated in the figure legends. l-Amino acid stock solutions were added to MOPS medium at the following final concentrations (mM): alanine (0.8), arginine (5.2), asparagine (0.4), aspartate (0.4), cysteine (0.1), glutamic acid (0.6), glutamine (0.6), glycine (0.8), histidine (0.2), isoleucine (0.4), leucine (0.8), lysine (0.4), methionine (0.2), phenylalanine (0.4), proline (0.4), serine (10.0), threonine (0.4), tryptophan (0.1), tyrosine (0.2), valine (0.6), and thiamine (0.01). When necessary, the following antibiotics were added to growth media: ampicillin (100 μg/ml), tetracycline (15 μg/ml), gentamicin (10 μg/ml), kanamycin (50 μg/ml), and chloramphenicol (25 μg/ml). Overnight cultures were routinely used to inoculate LB broth or minimal medium unless otherwise indicated. For strains carrying a *dps* deletion, exponentially growing cultures were used to prepare frozen glycerol stocks and to inoculate LB broth to eliminate/minimize the time spent under stationary-phase conditions.

10.1128/mBio.01034-19.2 TABLE S2 Strains, plasmids, and bacteriophage used in this study. Download Table S2, DOCX file, 0.1 MB. Copyright © 2019 Pourciau et al. 2019 Pourciau et al. This content is distributed under the terms of the Creative Commons Attribution 4.0 International license.

Transduction with P1*vir* was used to introduce gene deletions and disruptions from E. coli donor strains constructed in previous studies (31, 80) and from the Keio library (81) (Table S2). Plasmids pBR322 (82) and pCRA16 (54) (*csrA* cloned into pBR322) were used in complementation tests. The Flp recombinase encoded in pCP20 (83) was used to eliminate the kanamycin resistance cassette, as required.

### Considerations for strain construction.

Because complete loss of CsrA activity causes severe growth defects and genetic instability (84, 85), our experiments were performed with strains carrying a mutation (*csrA*::*gm*) producing a protein that contains the first 50 of 61 amino acids of the native protein and has residual CsrA activity (31). This *csrA* allele can be transduced by selection for a distally encoded gentamicin resistance (*gm*) marker. Strains containing *fur csrA* double mutations produced copious biofilm, which interfered with growth and reporter assays (data not shown). CsrA represses *pgaABCD* expression, required for synthesis and secretion of the biofilm adhesin poly-β-1,6-*N*-acetyl-d-glucosamine (PGA) (54), and studies have also documented a role for Fur in biofilm formation (86, 87). Deletion of *pgaC*, encoding the PGA glycosyl transferase, abolished biofilm production by the strains in LB medium. Consequently, studies in LB were performed in the *pgaC* mutant background.

### Construction of ′*lacZ* reporter fusions.

Chromosomal translational fusions to ′*lacZ* were constructed using the CRIM system (88) and plasmid vector pLFT (4) and integrated at the λ*att* site. Single-copy integrants were confirmed by PCR, as described previously (88). Constructions were performed as follows: ∼500 nucleotides (nt) of DNA upstream of and including the promoter region through one or more codons downstream of the translational start site was amplified by PCR using the associated primers (see Table S3). The PCR products were gel purified, digested with PstI and BamHI, ligated into PstI- and BamHI-digested and dephosphorylated plasmid pLFT, and electroporated into DH5α λpir cells. The fusion sequences were verified, and plasmids were isolated and integrated into the λ*att* site of strain MG1655 Δ*lacZ* using the helper plasmid pFINT (4).

10.1128/mBio.01034-19.3 TABLE S3 List of primers used in this study. Download Table S3, DOCX file, 0.1 MB. Copyright © 2019 Pourciau et al. 2019 Pourciau et al. This content is distributed under the terms of the Creative Commons Attribution 4.0 International license.

### β-Galactosidase assay.

Strains containing ′*lacZ* fusions were grown at 37°C in Luria-Bertani (LB) broth. Tetracycline (15 μg/ml) was used to ensure plasmid maintenance in strains bearing pBR322 or pCRA16. Cells were harvested at various times and β-galactosidase activity was measured as described previously (4). Total cell protein was measured following precipitation with 10% trichloroacetic acid, using the bicinchoninic acid (BCA) assay (Pierce Biotechnology) with bovine serum albumin as the protein standard.

### Electrophoretic gel mobility shift assays for RNA binding.

The binding of CsrA to *ftnB*, *dps*, and *bfr* transcripts was determined by EMSA using *in vitro*-synthesized *ftnB*, *dps*, and *bfr* transcripts (MAXIscript SP6/MEGAshortscript T7 kits; Ambion) and recombinant CsrA-His_6_ (14). The template DNAs for *in vitro* transcription of *ftnB* and *bfr* were generated by PCR from MG1655 genomic DNA, using oligonucleotide pairs *ftnB* SP6 fwd EMSA/*ftnB* SP6 rev EMSA and *bfr* T7 fwd EMSA/*bfr* T7 rev EMSA. The template DNA for *in vitro* transcription of *dps* was generated by annealing the oligonucleotide pair *dps* T7 EMSA/*dps* T7 EMSA comp. RNA was synthesized from the *ftnB* (127 nt, consisting of 127 nt of the 5′ UTR), *dps* (50 nt, consisting of 39 nt of the 5′ UTR and 11 nt of the coding region), and *bfr* (81 nt, consisting of the 23 nt 5′ UTR and 58 nt of the coding region) templates *in vitro* using the MEGAshortscript kit (Ambion). The resulting transcripts were purified via denaturing polyacrylamide gel electrophoresis (PAGE) followed by phenol-chloroform extraction and ethanol precipitation. Transcripts were treated with Antarctic phosphatase (NEB) and radiolabeled at the 5′ end using [γ-^32^P]ATP and T4 polynucleotide kinase. Binding reaction mixtures contained 0.5 nM RNA, 10 mM MgCl_2_, 100 mM KCl, 32.5 ng total yeast RNA, 20 mM dithiothreitol (DTT), 7.5% glycerol, 4 U SUPERasin (Ambion), and various concentrations of recombinant CsrA and were incubated at 37°C for 30 min. Reaction mixtures were separated on 9% native polyacrylamide gels (for *ftnB* mRNA) and 12% native polyacrylamide gels (for *dps* and *bfr* mRNAs) with 1× Tris-borate-EDTA (TBE) buffer. Competition assays were performed in the presence or absence of unlabeled specific (self) and nonspecific (*phoB*) RNA competitors using the minimum CsrA concentration required for a full shift. Labeled RNA was analyzed using a phosphorimager equipped with Quantity One software (Bio-Rad), as previously described (4). The apparent equilibrium binding constant (*K_D_*) for CsrA-RNA complex formation was calculated according to a previously described cooperative binding equation (14).

### Footprint assay.

CsrA-*ftnB* RNA footprint assays were performed according to a published procedure (24). *ftnB* RNA (nt +1 to +152) was synthesized with the RNAMaxx kit (Agilent Technologies) using PCR-generated DNA templates. Gel-purified RNA was dephosphorylated and then 5′ end labeled using T4 polynucleotide kinase (New England BioLabs) and [γ-^32^P]ATP (7,000 Ci/mmol). Labeled RNAs were renatured by heating for 1 min at 90°C followed by slow cooling to room temperature. Binding reaction mixtures (10 μl) contained 2 nM labeled RNA, 10 mM Tris-HCl (pH 7.5), 10 mM MgCl_2_, 100 mM KCl, 40 ng of yeast RNA, 7.5% glycerol, 0.1 mg/ml xylene cyanol, and various concentrations of purified CsrA-His_6_. After a 30-min incubation at 37°C to allow for CsrA-RNA complex formation, RNase T1 (0.016 U) was added and the incubation was continued for 15 min at 37°C. The reactions were stopped by adding 10 μl of stop solution (95% formamide, 0.025% SDS, 20 mM EDTA, 0.025% bromophenol blue, 0.025% xylene cyanol). Samples were heated for 5 min at 90°C and fractionated through standard 6% (vol/vol) polyacrylamide-8 M urea sequencing gels. Cleaved patterns were examined using a Typhoon 8600 variable mode imager.

### Coupled transcription-translation assay.

*In vitro* coupled transcription-translation assays using PURExpress (New England BioLabs) were performed according to a published procedure (89). Plasmid pYH333 contains a T7 promoter driving transcription of the *bfr* translational fusion (nt +1 to +50 relative to the *bfr* transcriptional start site). Plasmid pYH334 contains a T7 promoter driving transcription of the *dps* translational fusion (nt +1 to +81 relative to the *dps* transcriptional start site). Plasmid pYH336 contains a T7 promoter driving transcription of the *ftnB* translational fusion (nt +1 to +152 relative to the *ftnB* transcriptional start site). These plasmids were used as the templates for coupled transcription-translation reactions using the PURExpress *in vitro* protein synthesis kit, according to the manufacturer's instructions. Each 6.7-μl reaction mixture contained 250 ng of plasmid DNA template, various concentrations of purified CsrA-H_6_, 1 U of RNasin (Promega), 2.5 mM DTT, 2.7 μl of solution A, and 2 μl of solution B. The mixtures were incubated for 2.5 h at 37°C, and β-galactosidase activity was determined according to the manufacturer’s instructions.

### Cellular iron measurement.

Total cellular Fe concentrations were measured by inductively coupled plasma optical emission spectrometry (ICP-OES) at the University of Florida Institute of Food and Agricultural Sciences Analytical Services Laboratories. LB medium (500 ml) was inoculated with overnight cultures and incubated at 37°C with shaking. Two hundred fifty milliliters of exponential-phase cultures (OD_600_ of 0.5) were harvested by centrifugation and washed three times in cold phosphate-buffered saline (PBS). The resulting bacterial pellets were resuspended in 1 ml 35% HNO_3_ (trace-metal grade) treated at 95°C and diluted 1:10 with Invitrogen UltraPure distilled water. Fe concentrations were normalized to total protein. For quantification of iron in the medium, 2 ml trace-metal-grade 35% HNO_3_ was added to 18 ml medium for ICP-OES analysis. Glassware for these experiments was pretreated overnight to remove trace metal contamination with 20% trace-metal-grade HNO_3_ and multiple rinses with Nanopure water.

### Growth kinetics assay.

Growth under Fe-limiting and -replete conditions was monitored by OD_600_ measurements in MOPS minimal medium (see “Bacterial strains and culture conditions”) or by BCA protein assay in Luria-Bertani (LB) medium. All medium components were filter sterilized. Prior to use, labware was treated overnight in a 20% trace-metal-grade HNO_3_ and rinsed multiple times with Nanopure water. ICP-OES analysis indicated that final Fe concentration of the MOPS medium before Fe supplementation was below 0.01 μM. For growth experiments performed in MOPS, overnight cultures were grown in MOPS with 0.05 μM FeSO_4_ and were diluted 1:100 (except for cultures carrying the *dps* mutation, which were inoculated directly from −80°C glycerol stocks) and grown to exponential phase in MOPS with 0.05 or 100 μM FeSO_4_. Growth experiments in LB were started from overnight cultures in LB, which were diluted 1:100 and grown to exponential phase in LB. The growth curves in all media were started at an OD_600_ of 0.01 from the exponentially growing cultures. Total cell protein was measured following precipitation with 10% trichloroacetic acid, using the BCA assay (Pierce Biotechnology) with bovine serum albumin as the protein standard. The growth rate constant (μ) was calculated from the exponential phase of growth: μ = 2.303(logOD_2_ − logOD_1_)/(*t*_2_ – *t*_1_).

### Western blotting of CsrA and FecB-3×FLAG protein.

Samples for CsrA immunoblotting were harvested at intervals after the addition of 250 μM iron chelator 2,2′‐dipyridyl. Samples for FecB-3×FLAG blots were harvested at an OD_600_ of 0.05 from LB supplemented with 1 mM sodium citrate to induce expression. Cells were centrifuged and resuspended in 2× sample buffer (4% [wt/vol] SDS, 0.16 M Tris, 1.5% [vol/vol] β-mercaptoethanol, 20% [vol/vol] glycerol, 0.02% [wt/vol] bromophenol blue, pH 6.0), normalized by the OD, and lysed by sonication and boiling. Samples were separated by SDS-PAGE, transferred to 0.2 μM polyvinylidene difluoride (PVDF) membranes, and detected using polyclonal anti-CsrA or monoclonal anti-FLAG for FecB-3×FLAG, or anti-RpoB antibodies, as described previously (90).

### Northern blotting.

Samples were harvested and total cellular RNA was isolated using the RNeasy minikit (Qiagen). Total RNA was mixed with 2 volumes of loading buffer (50% [vol/vol] deionized formamide, 6% [vol/vol] formaldehyde, 1× MOPS [20 mM], 5 mM sodium acetate [NaOAc], 2 mM EDTA [pH 7.0], 10% [vol/vol] glycerol, 0.05% [wt/vol] bromophenol blue, 0.01% [wt/vol] ethidium bromide), denatured by heating at 95°C for 5 min, placed on ice, and separated by electrophoresis on a 7 M urea-5% polyacrylamide gel. RNA was transferred and fixed to a positively charged nylon membrane (Roche) and hybridized with digoxigenin (DIG)-labeled antisense CsrB, CsrC, or RyhB RNA probes as previously described (5). Transferred rRNA served as a loading control and was stained with methylene blue, imaged using a Gel-Doc, and signal intensity was quantified using Quantity One software. CsrB or CsrC RNAs signals were captured with a ChemiDoc XRS+ system (Bio-Rad, Hercules, CA).

### Oxidative stress assay.

WT and mutant strains were grown in LB to mid-exponential (OD_600_ of 0.5) or early stationary (OD_600_ of 2.5) phase and exposed to H_2_O_2_ (12.5 mM and 50.0 mM, respectively). After 0, 15, and 30 min, samples were collected, washed 3 times in pH 7.4 PBS, and 10-fold serially diluted. Cells were plated on LB and grown for 18 h at 37°C before imaging the resulting colonies.
