# Supplementary material for: Regulation of Iron Storage by CsrA Supports Exponential Growth of Escherichia coli
Source: mBio. 2019 Aug 6;10(4):e01034-19. doi: 10.1128/mBio.01034-19 (PMC6686035; doi:10.1128/mBio.01034-19)
Supplement: TABLE S1 [file mBio.01034-19-st001.docx]

| **Gene** | **Protein Function** | **Genes in Operon** | **Evidence for CsrA Regulation** | **Evidence for CsrA Binding** |
| --- | --- | --- | --- | --- |
| *fecB* | Ferric citrate ABC transporter periplasmic binding protein | *fecABCDE* | RPF | CO, CLIP |
| *fepA* | Outer membrane transporter for ferrienterobactin | *fepA, entD* | RPF, RNA |  |
| *fhuA* | Outer membrane transporter for ferrichrome | *fhuABCD* | RPF, RNA |  |
| *fhuE* | Outer membrane transporter for coprogen, ferrioxamine B, and rhodotorulic acid | *fhuE* | RPF, RNA |  |
| *dps* | DNA protection during starvation protein; Fe- and DNA binding, DNA protection | *dps* | RPF, RNA, decay | CO |
| *ftnA* | Ferritin A, ferritin iron storage protein (cytoplasmic) | *ftnA* | RPF, RNA |  |
| *ftnB* | Ferritin B, ferritin-like iron binding protein | *ftnB* | RPF, RNA, decay |  |
| *bfr* | Bacterioferritin; iron-storage iron storage protein that can bind haem cofactors | *bfr* | RPF, RNA | PW |
| *fes* | Enterobactin esterase | *fes, entF, fepE* | RPF, RNA |  |
| *entC* | Enterobactin biosynthesis | *entCEBAH* | RPF, RNA |  |
| *sufA* | Fe-S cluster assembly scaffold | *sufABCDSE* | RPF, RNA |  |
| *fur* | Ferric iron uptake global transcriptional repressor | *fur* |  |  |

Historical evidence of CsrA effects on genes related to iron metabolism.

Evidence for CsrA regulation: RPF = Ribosome Protected Fragments (1), RNA = steady state RNA abundance (1), decay = RNA half-life (1). Evidence for CsrA Binding: CLIP = CsrA-RNA CLIP-seq (1), CO = RNA copurified with CsrA-His_6_ (2), PW = position weight matrix analysis CsrA target prediction study (3).

**REFERENCES**

1. Potts AH, Vakulskas CA, Pannuri A, Yakhnin H, Babitzke P, Romeo T. 2017. Global role of the bacterial post-transcriptional regulator CsrA revealed by integrated transcriptomics. Nat Commun 8:1596.

2. Edwards AN, Patterson-Fortin LM, Vakulskas CA, Mercante JW, Potrykus K, Vinella D, Camacho MI, Fields JA, Thompson SA, Georgellis D, Cashel M, Babitzke P, Romeo T. 2011. Circuitry linking the Csr and stringent response global regulatory systems. Mol Microbiol 80:1561-80.

3. Kulkarni PR, Jia T, Kuehne SA, Kerkering TM, Morris ER, Searle MS, Heeb S, Rao J, Kulkarni RV. 2014. A sequence-based approach for prediction of CsrA/RsmA targets in bacteria with experimental validation in *Pseudomonas aeruginosa*. Nucleic Acids Res 42:6811-25.
