# Supplementary figures and images for: Regulation of Iron Storage by CsrA Supports Exponential Growth of Escherichia coli
Source: mBio. 2019 Aug 6;10(4):e01034-19. doi: 10.1128/mBio.01034-19 (PMC6686035; doi:10.1128/mBio.01034-19)

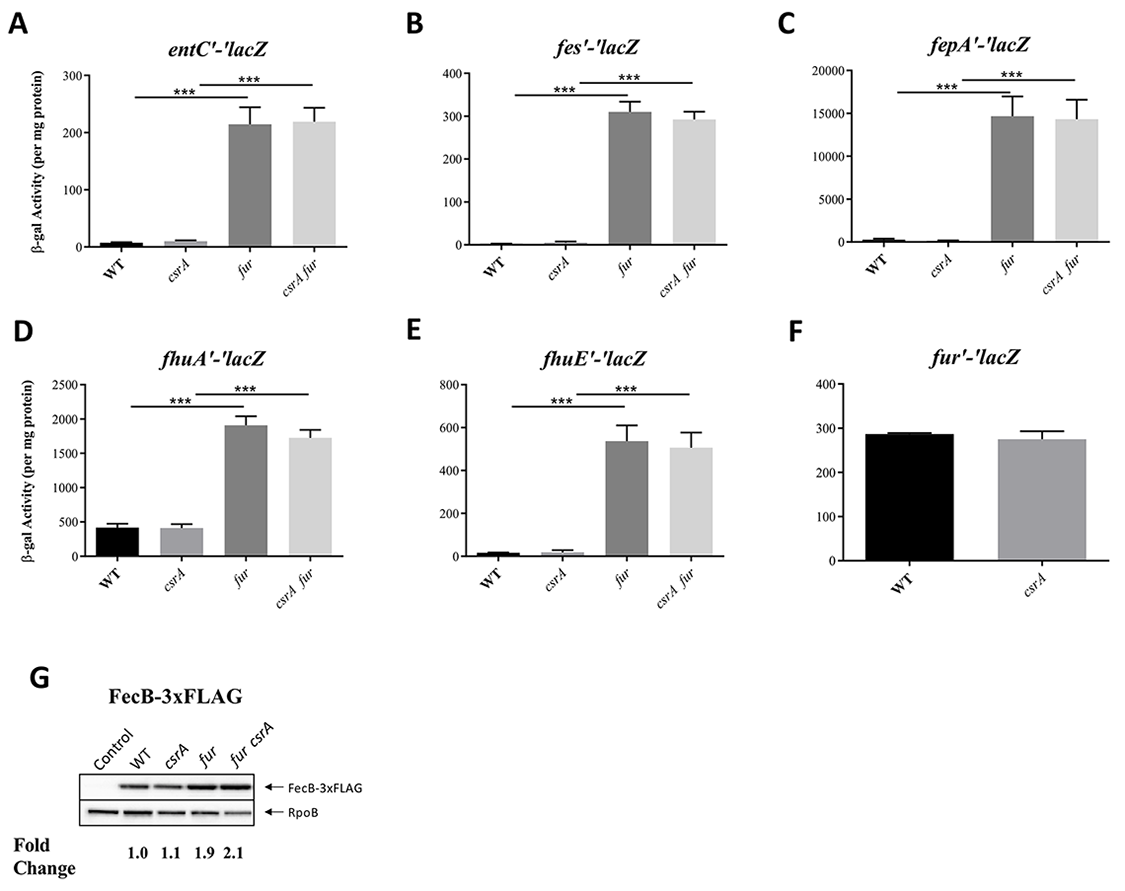

Supplement: FIG S1 [file mBio.01034-19-sf001.tif]

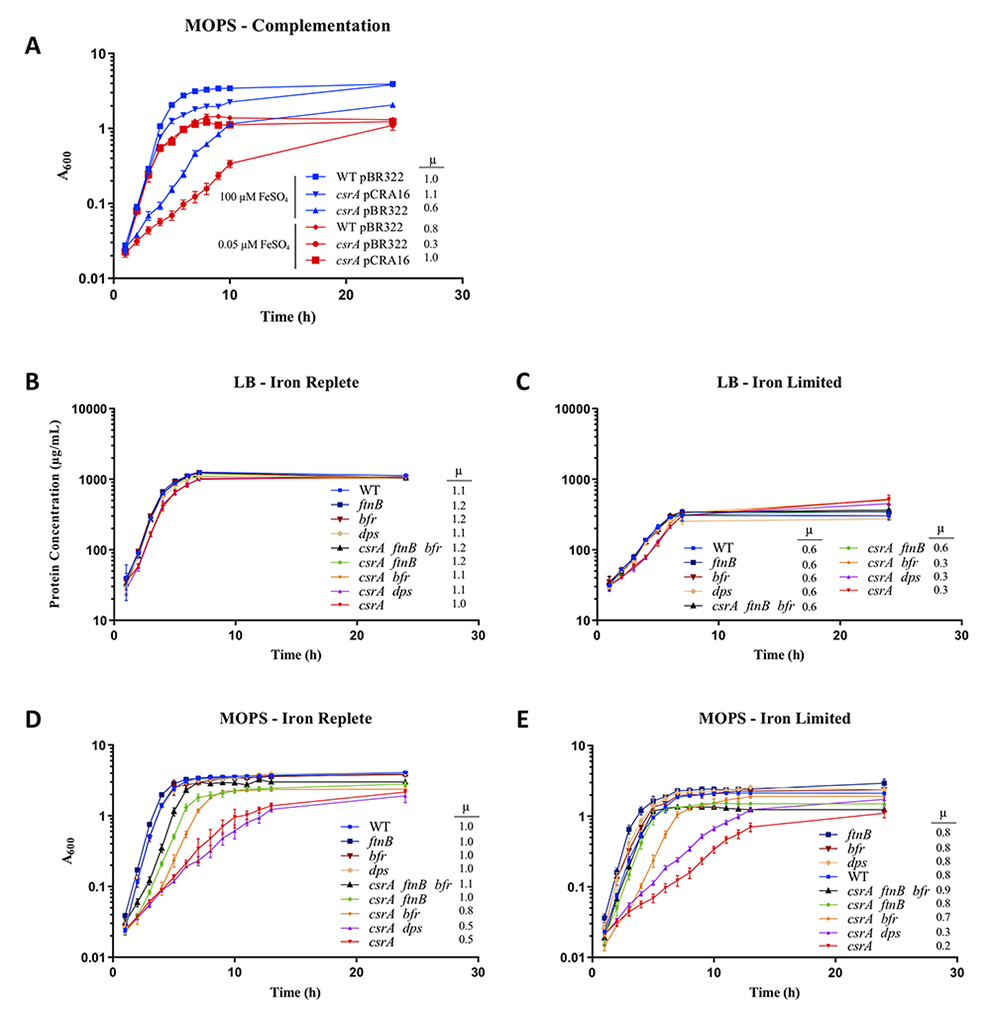

Supplement: FIG S2 [file mBio.01034-19-sf002.tif]

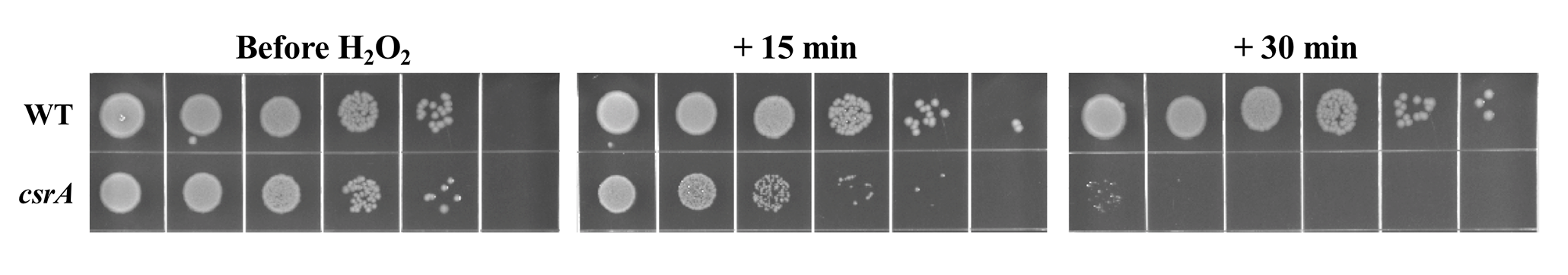

Supplement: FIG S3 [file mBio.01034-19-sf003.tif]
