# Supplementary material for: Regulation of Iron Storage by CsrA Supports Exponential Growth of Escherichia coli
Source: mBio. 2019 Aug 6;10(4):e01034-19. doi: 10.1128/mBio.01034-19 (PMC6686035; doi:10.1128/mBio.01034-19)
Supplement: TABLE S2 [file mBio.01034-19-st002.docx]

| *Name* | *Genotype or description* | *Reference or Source* |
| --- | --- | --- |
| *E. coli strains* |  |  |
| MG1655 | F^−^ λ^−^ rph-1 | CGSC (no. 6300) |
| AP379 | MG1655 ΔlacZ | (1) |
| MG1655 *csrA::gm* | MG1655 with *csrA* disrupted after amino acid position 50 - Gm^r^ | (2) |
| XWC880 | MG1655 with marked *pgaC* disruption-Cam^r^ | (3) |
| BW25113 Δ*fur::kan* | BW25113 with marked *fur* deletion-Kan^r^ | (4) |
| BW25113 Δ*ftnB::kan* | BW25113 with marked *ftnB* deletion-Kan^r^ | (4) |
| BW25113 Δ*dps::kan* | BW25113 with marked *dps* deletion-Kan^r^ | (4) |
| BW25113 Δ*bfr::kan* | BW25113 with marked *bfr* deletion-Kan^r^ | (4) |
| BW25113 Δ*csgA::kan* | BW25113 with marked *csgA* deletion-Kan^r^ | (4) |
| MG1655 Δ*csrB::kan* | MG1655 with marked *csrB* deletion-Kan^r^ | (5) |
| MG1655 Δ*csrC::kan* | MG1655 with marked *csrC* deletion-Kan^r^ | (5) |
| CP001 | AP379 pLFT*fhuA′-′lacZ* | This study |
| CP002 | AP379 pLFT*fhuE′-′lacZ* | This study |
| CP003 | AP379 pLFT*fes′-′lacZ* | This study |
| CP004 | AP379 pLFT*sufA′-′lacZ* | This study |
| CP005 | AP379 pLFT*dps′-′lacZ* | This study |
| CP006 | AP379 pLFT*ftnA′-′lacZ* | This study |
| CP007 | AP379 pLFT*ftnB′-′lacZ* | This study |
| CP008 | AP379 pLFT*bfr′-′lacZ* | This study |
| CP009 | AP379 pLFT*entC′-′lacZ* | This study |
| CP010 | AP379 pLFT*fur′-′lacZ* | This study |
| CP011 | AP379 pLFT*fepA′-′lacZ* | This study |
| CP012 | MG1655 *fecB*::3×FLAG | This study |
| CP013 | AP379 pLFT*fhuA′-′lacZ pgaC::cam* | This study |
| CP014 | AP379 pLFT*fhuE′-′lacZ pgaC::cam* | This study |
| CP015 | AP379 pLFT*fes′-′lacZ pgaC::cam* | This study |
| CP016 | AP379 pLFT*sufA′-′lacZ pgaC::cam* | This study |
| CP017 | AP379 pLFT*dps′-′lacZ pgaC::cam* | This study |
| CP018 | AP379 pLFT*ftnA′-′lacZ pgaC::cam* | This study |
| CP019 | AP379 pLFT*ftnB′-′lacZ pgaC::cam* | This study |
| CP020 | AP379 pLFT*bfr′-′lacZ pgaC::cam* | This study |
| CP021 | AP379 pLFT*entC′-′lacZ pgaC::cam* | This study |
| CP022 | AP379 pLFT*fur′-′lacZ pgaC::cam* | This study |
| CP023 | AP379 pLFT*fepA′-′lacZ pgaC::cam* | This study |
| CP024 | MG1655 *fecB*::3×FLAG *pgaC::cam* | This study |
| CP025 | AP379 pLFT*fhuA′-′lacZ pgaC::cam csrA::gm* | This study |
| CP026 | AP379 pLFT*fhuE′-′lacZ pgaC::cam csrA::gm* | This study |
| CP027 | AP379 pLFT*fes′-′lacZ pgaC::cam csrA::gm* | This study |
| CP028 | AP379 pLFT*sufA′-′lacZ pgaC::cam csrA::gm* | This study |
| CP029 | AP379 pLFT*dps′-′lacZ pgaC::cam csrA::gm* | This study |
| CP030 | AP379 pLFT*ftnA′-′lacZ pgaC::cam csrA::gm* | This study |
| CP031 | AP379 pLFT*ftnB′-′lacZ pgaC::cam csrA::gm* | This study |
| CP032 | AP379 pLFT*bfr′-′lacZ pgaC::cam csrA::gm* | This study |
| CP033 | AP379 pLFT*entC′-′lacZ pgaC::cam csrA::gm* | This study |
| CP034 | AP379 pLFT*fur′-′lacZ pgaC::cam csrA::gm* | This study |
| CP035 | AP379 pLFT*fepA′-′lacZ pgaC::cam csrA::gm* | This study |
| CP036 | MG1655 *fecB*::3×FLAG *pgaC::cam csrA::gm* | This study |
| CP037 | AP379 pLFT*fhuA′-′lacZ pgaC::cam* Δ*fur::kan* | This study |
| CP038 | AP379 pLFT*fhuE′-′lacZ pgaC::cam* Δ*fur::kan* | This study |
| CP039 | AP379 pLFT*fes′-′lacZ pgaC::cam* Δ*fur::kan* | This study |
| CP040 | AP379 pLFT*sufA′-′lacZ pgaC::cam* Δ*fur::kan* | This study |
| CP041 | AP379 pLFT*dps′-′lacZ pgaC::cam* Δ*fur::kan* | This study |
| CP042 | AP379 pLFT*ftnA′-′lacZ pgaC::cam* Δ*fur::kan* | This study |
| CP043 | AP379 pLFT*ftnB′-′lacZ pgaC::cam* Δ*fur::kan* | This study |
| CP044 | AP379 pLFT*bfr′-′lacZ pgaC::cam* Δ*fur::kan* | This study |
| CP045 | AP379 pLFT*entC′-′lacZ pgaC::cam* Δ*fur::kan* | This study |
| CP046 | AP379 pLFT*fur′-′lacZ pgaC::cam* Δ*fur::kan* | This study |
| CP047 | AP379 pLFT*fepA′-′lacZ pgaC::cam* Δ*fur::kan* | This study |
| CP048 | MG1655 *fecB*::3×FLAG *pgaC::cam* Δ*fur::kan* | This study |
| CP050 | AP379 pLFT*fhuA′-′lacZ pgaC::cam csrA::gm* Δ*fur::kan* | This study |
| CP051 | AP379 pLFT*fhuE′-′lacZ pgaC::cam csrA::gm* Δ*fur::kan* | This study |
| CP052 | AP379 pLFT*fes′-′lacZ pgaC::cam csrA::gm* Δ*fur::kan* | This study |
| CP053 | AP379 pLFT*sufA′-′lacZ pgaC::cam csrA::gm* Δ*fur::kan* | This study |
| CP054 | AP379 pLFT*dps′-′lacZ pgaC::cam csrA::gm* Δ*fur::kan* | This study |
| CP055 | AP379 pLFT*ftnA′-′lacZ pgaC::cam csrA::gm* Δ*fur::kan* | This study |
| CP056 | AP379 pLFT*ftnB′-′lacZ pgaC::cam csrA::gm* Δ*fur::kan* | This study |
| CP057 | AP379 pLFT*bfr′-′lacZ pgaC::cam csrA::gm* Δ*fur::kan* | This study |
| CP058 | AP379 pLFT*entC′-′lacZ pgaC::cam csrA::gm* Δ*fur::kan* | This study |
| CP059 | AP379 pLFT*fur′-′lacZ pgaC::cam csrA::gm* Δ*fur::kan* | This study |
| CP060 | AP379 pLFT*fepA′-′lacZ pgaC::cam csrA::gm* Δ*fur::kan* | This study |
| CP061 | MG1655 *fecB*::3×FLAG *pgaC::cam csrA::gm* Δ*fur::kan* | This study |
| CP062 | AP379 pLFT*dps′-′lacZ pgaC::cam* pBR322 Tet^r^ Amp^r^ | This study |
| CP063 | AP379 pLFT*dps′-′lacZ pgaC::cam csrA::gm* pBR322 Tet^r^ Amp^r^ | This study |
| CP064 | AP379 pLFT*dps′-′lacZ pgaC::cam* *csrA::gm* pCRA16 Tet^r^ | This study |
| CP065 | AP379 pLFT*ftnB′-′lacZ pgaC::cam* pBR322 Tet^r^ Amp^r^ | This study |
| CP066 | AP379 pLFT*ftnB′-′lacZ pgaC::cam csrA::gm* pBR322 Tet^r^ Amp^r^ | This study |
| CP067 | AP379 pLFT*ftnB′-′lacZ pgaC::cam csrA::gm* pCRA16 Tet^r^ | This study |
| CP068 | AP379 pLFT*bfr′-′lacZ pgaC::cam* pBR322 Tet^r^ Amp^r^ | This study |
| CP069 | AP379 pLFT*bfr′-′lacZ pgaC::cam csrA::gm* pBR322 Tet^r^ Amp^r^ | This study |
| CP070 | AP379 pLFT*bfr′-′lacZ pgaC::cam csrA::gm* pCRA16 Tet^r^ | This study |
| CP071 | MG1655 *pgaC::cam* pBR322 Tet^r^ Amp^r^ | This study |
| CP072 | MG1655 *pgaC::cam* pCRA16 Tet^r^ | This study |
| CP073 | MG1655 *pgaC::cam csrA::gm* pBR322 Tet^r^ Amp^r^ | This study |
| CP074 | MG1655 *pgaC::cam csrA::gm* pCRA16 Tet^r^ | This study |
| CP075 | MG1655 *pgaC::cam* Δ*ftnB::kan* | This study |
| CP076 | MG1655 *pgaC::cam* Δ*bfr::kan* | This study |
| CP077 | MG1655 *pgaC::cam* Δ*dps::kan* | This study |
| CP078 | MG1655 *pgaC::cam* *csrA::gm* Δ*ftnB::kan* | This study |
| CP079 | MG1655 *pgaC::cam* *csrA::gm* Δ*bfr::kan* | This study |
| CP080 | MG1655 *pgaC::cam* *csrA::gm* Δ*dps::kan* | This study |
| CP081 | MG1655 *pgaC::cam* *csrA::gm* Δ*ftnB* Δ*bfr::kan* | This study |
| CP082 | MG1655 *pgaC::cam* *csrA::gm* Δ*ftnB* Δ*dps::kan* | This study |
| CP083 | MG1655 *pgaC::cam* *csrA::gm* Δ*bfr* Δ*dps::kan* | This study |
| CP084 | MG1655 Δ*csgA::kan* | This study |
| CP085 | MG1655 Δ*csgA::kan* pBR322 Tet^r^ Amp^r^ | This study |
| CP086 | MG1655 Δ*csgA::kan csrA::gm* pBR322 Tet^r^ Amp^r^ | This study |
| CP087 | MG1655 Δ*csgA::kan csrA::gm* pCRA16 Tet^r^ | This study |
| CP088 | MG1655 Δ*csgA* Δ*ftnB::kan* | This study |
| CP089 | MG1655 Δ*csgA* Δ*bfr::kan* | This study |
| CP090 | MG1655 Δ*csgA* Δ*dps::kan* | This study |
| CP091 | MG1655 Δ*csgA::kan* *csrA::gm* | This study |
| CP092 | MG1655 Δ*csgA csrA::gm* Δ*ftnB::kan* | This study |
| CP093 | MG1655 Δ*csgA* *csrA::gm* Δ*bfr::kan* | This study |
| CP094 | MG1655 Δ*csgA* *csrA::gm* Δ*dps::kan* | This study |
| CP095 | MG1655 Δ*csgA csrA::gm* Δ*ftnB* Δ*dps::kan* | This study |
| CP095 | MG1655 Δ*csgA csrA::gm* Δ*ftnB* Δ*dps::kan* | This study |
| CP096 | MG1655 Δ*csgA csrA::gm* Δ*ftnB* Δ*bfr::kan* | This study |
| CP097 | MG1655 Δ*csgA csrA::gm* Δ*bfr* Δ*dps::kan* | This study |
| *Plasmids* |  |  |
| pLFT | Used for constructing translational fusions; Amp^r^ | (1) |
| pPFINT | Helper plasmid used for integrating *lacZ* fusions into the chromosome; Tet^r^ | (1) |
| pCP20 | Used for eliminating antibiotic resistance genes; Tet^r^ | (6) |
| pBR322 | Cloning vector; Tet^r^ Amp^r^ | (7) |
| pCRA16 | *csrA* gene from K-12 cloned into blunt-ended VspI site of *bla* of pBR322; Tet^r^ | (8) |
| pHP4 | *pnp* control for PURExpress | (9) |
| pYH333 | *bfr* under T7 promoter from +1 to +50 in pLFT vector for PURExpress | This study |
| pYH334 | *dps* under T7 promoter from +1 to +81 in pLFT vector for PURExpress | This study |
| pYH336 | *ftnB* under T7 promoter from +1 to +152 in pLFT vector for PURExpress | This study |
| *Bacteriophage* |  |  |
| P1vir | Strictly lytic P1 | Carol Gross |

Strains, plasmids, and bacteriophage used in this study.

**REFERENCES**

1. Pannuri A, Vakulskas CA, Zere T, McGibbon LC, Edwards AN, Georgellis D, Babitzke P, Romeo T. 2016. Circuitry linking the catabolite repression and Csr global regulatory systems of *Escherichia coli*. J Bacteriol 198:3000-3015.

2. Vakulskas CA, Leng Y, Abe H, Amaki T, Okayama A, Babitzke P, Suzuki K, Romeo T. 2016. Antagonistic control of the turnover pathway for the global regulatory sRNA CsrB by the CsrA and CsrD proteins. Nucleic Acids Res 44:7896-910.

3. Wang X, Preston JF, Romeo T. 2004. The *pgaABCD* locus of *Escherichia coli* promotes the synthesis of a polysaccharide adhesin required for biofilm formation. J Bacteriol 186:2724-34.

4. Baba T, Ara T, Hasegawa M, Takai Y, Okumura Y, Baba M, Datsenko KA, Tomita M, Wanner BL, Mori H. 2006. Construction of *Escherichia coli* K-12 in-frame, single-gene knockout mutants: the Keio collection. Mol Syst Biol 2:2006.0008.

5. Zere TR, Vakulskas CA, Leng Y, Pannuri A, Potts AH, Dias R, Tang D, Kolaczkowski B, Georgellis D, Ahmer BM, Romeo T. 2015. Genomic targets and features of BarA-UvrY (-SirA) signal transduction systems. PLoS One 10:e0145035.

6. Cherepanov PP, Wackernagel W. 1995. Gene disruption in *Escherichia coli*: TcR and KmR cassettes with the option of Flp-catalyzed excision of the antibiotic-resistance determinant. Gene 158:9-14.

7. Sircili MP, Walters M, Trabulsi LR, Sperandio V. 2004. Modulation of enteropathogenic *Escherichia coli* virulence by quorum sensing. Infect Immun 72:2329-37.

8. Wang X, Dubey AK, Suzuki K, Baker CS, Babitzke P, Romeo T. 2005. CsrA post-transcriptionally represses *pgaABCD*, responsible for synthesis of a biofilm polysaccharide adhesin of *Escherichia coli*. Mol Microbiol 56:1648-63.

9. Park H, Yakhnin H, Connolly M, Romeo T, Babitzke P. 2015. CsrA Participates in a PNPase Autoregulatory Mechanism by Selectively Repressing Translation of *pnp* Transcripts That Have Been Previously Processed by RNase III and PNPase. J Bacteriol 197:3751-9.
