# Supplementary material for: Regulation of Iron Storage by CsrA Supports Exponential Growth of Escherichia coli
Source: mBio. 2019 Aug 6;10(4):e01034-19. doi: 10.1128/mBio.01034-19 (PMC6686035; doi:10.1128/mBio.01034-19)
Supplement: TABLE S3 [file mBio.01034-19-st003.docx]

| *Oligonucleotide* | | *Sequence* | *Purpose* |
| --- | --- | --- | --- |
| *bfr’-‘lacZ* fwd | 5' GAGGAGCTGCAGAACCTGTCTCCGGAATTCCACGGC 3' | | Forward primer for constructing translational fusion |
| *bfr’-‘lacZ* rev | 5' CTCCTCGGATCCACAAGCTCATTTCCCAACAGTTTGTTGAG 3' | | Reverse primer for constructing translational fusion |
| *bfr* conf fwd | 5’ GCCGGAGTTTAAGGAGTCC 3’ | | Forward primer for confirming *bfr* mutant |
| *bfr* conf rev | 5’ GCTCGGCAGCATGATAAACC 3’ | | Reverse primer for confirming *bfr* mutant |
| *bfr* T7 fwd EMSA | 5’ TAATACGACTCACTATAGGGTGGAAGCGAAGGAGTCAAAAAA TGAAAGGTG 3’ | | Oligo for RNA transcription |
| *bfr* T7 rev EMSA | 5’ ACAAGCTCATTTCCCAACAGTTTGTTGAGATAATTTATAAC 3’ | | Oligo for RNA transcription |
| *csrA* conf fwd | 5’ ATGTAATGCCATGACTGCTTAGATG 3’ | | Forward primer for confirming *csrA* mutant |
| *csrA* conf rev | 5’ TAAGTTGAATGAACGGGAGTAAAGC 3’ | | Reverse primer for confirming *csrA* mutant |
| *csrB*R | 5’ GCGTTAAAGGACACCTCCAGG 3’ | | Forward primer for *csrB* northern probe |
| *csrB*T7 | 5’ GTAATACGACTCACTATAGGTTCGTTTCGCAGCATTCCAG 3’ | | Reverse primer for *csrB* northern probe |
| *csrC*R | 5’ GAGGACGCTAACAGGAACAATG 3’ | | Forward primer for *csrC* northern probe |
| *csrC*T7 | 5’ GTAATACGACTCACTATAGGTCTTACAATCCTTGCAGGC 3’ | | Reverse primer for *csrC* northern probe |
| *dps’-‘lacZ* fwd | 5' GAGGAGCTGCAGTCTCTCGACCGCTCTGCCTTA 3 | | Forward primer for constructing translational fusion |
| *dps’-‘lacZ* rev | 5' CTCCTCGGATCCGCGGTACTCATAATTTCATATCCTCTTGA 3 | | Reverse primer for constructing translational fusion |
| *dps* conf fwd | 5’ CCACTATTAGTGTGATAGGAACAGCC 3’ | | Forward primer for confirming *dps* mutant |
| *dps* conf rev | 5’ CACTAAATAAGTGCGTTGAGGTGGG 3’ | | Reverse primer for confirming *dps* mutant |
| *dps* T7 EMSA | 5’TAATACGACTCACTATAGGGTTAATTACTGGGACATAACATCAAGAGGATATGAAATTATGAGTACCGC 3’ | | Oligo for RNA transcription |
| *dps* T7 EMSA comp | 5’GCGGTACTCATAATTTCATATCCTCTTGATGTTATGTCCCAGTAATTAACCCTATAGTGAGTCGTATTA 3’ | | Oligo for RNA transcription |
| *entC’-‘lacZ* fwd | 5' GAGGAGCTGCAGCAGTGAGCCGGTCAGGGTGAC 3' | | Forward primer for constructing translational fusion |
| *entC’-‘lacZ* rev | 5' CTCCTCGGATCCTCTGCTGTACTTCCTCAGCCAGTGA 3' | | Reverse primer for constructing translational fusion |
| *fecA’-‘lacZ* fwd | 5' GAGGAGTCTAGATGCAACGCCCCTTCCGTGTCCTG 3' | | Forward primer for constructing translational fusion |
| *fecA’-‘lacZ* rev | 5' CTCCTCGAATTCAAACGCGTAACGGCGTCATACCTTCCCC 3' | | Reverse primer for constructing translational fusion |
| *fepA’-‘lacZ* fwd | 5’ GAGGAGCTGCAGAGTGCAGAAACAGCGTGCCCTC 3’ | | Forward primer for constructing translational fusion |
| *fepA’-‘lacZ* rev | 5’ GAGCACGGATCCCCATAAATCCCCAGATTGACCAACAAGGC 3’ | | Reverse primer for constructing translational fusion |
| *fes’-‘lacZ* fwd | 5' GAGGAGCTGCAGGTATCGCGCTCGCCACGC 3' | | Forward primer for constructing translational fusion |
| *fes’-‘lacZ* rev | 5' CTCCTCGGATCCCAGCTCTCACTTCCTACTTTTAACGCCGT 3' | | Reverse primer for constructing translational fusion |
| *fhuA’-‘lacZ* fwd | 5' GAGGAGCTGCAG GGGCAATGTCGATTTATCAGCGTT 3' | | Forward primer for constructing translational fusion |
| *fhuA’-‘lacZ* rev | 5' CTCCTCGGATCCGTTTTGGAACGCGCCATTGGTATATC 3' | | Reverse primer for constructing translational fusion |
| *fhuE’-‘lacZ* fwd | 5' GAGGAGCTGCAGTTCATGATCAGACGATAGCCATCT 3' | | Forward primer for constructing translational fusion |
| *fhuE’-‘lacZ* rev | 5' CTCCTCGGATCCTGATATTGATTATCCCTGTTAAATTGTGT 3' | | Reverse primer for constructing translational fusion |
| *ftnA’-‘lacZ* fwd | 5' GAGGAGCTGCAGCGCGTAAAATAGTGCTTTCTCTTACTC 3' | | Forward primer for constructing translational fusion |
| *ftnA’-‘lacZ* rev | 5' CTCCTCGGATCCGAAGAGTACAGTTCCAGGTTCATCTG 3' | | Reverse primer for constructing translational fusion |
| *ftnB’-‘lacZ* fwd | 5' GAGGAGCTGCAGCAGCAGATTAATCCATAAGATTAGCCTGG 3' | | Forward primer for constructing translational fusion |
| *ftnB’-‘lacZ* rev | 5' CTCCTCGGATCCTTGAGAAGCATTCCAGCGGTTGC 3' | | Reverse primer for constructing translational fusion |
| *ftnB* conf fwd | 5’ ATTTCGGACCGGCAGAAAGG 3’ | | Forward primer for confirming *ftnB* mutant |
| *ftnB* conf rev | 5’ TTGAACTCAAGTTTACCCCACAGG 3’ | | Reverse primer for confirming *ftnB* mutant |
| *ftnB* SP6 fwd EMSA | 5’ ATTTAGGTGACACTATAGAAGATCATCGTCAGAATTGACTCCACG3’ | | Oligo for RNA transcription |
| *ftnB* SP6 rev EMSA | 5’ ACCTAATATCCTTATATCCAGAAGT 3’ | | Oligo for RNA transcription |
| *fur’-‘lacZ* fwd | 5’ GAGGAGCTGCAGTCTCGAAGAGATTGATTTCAACGGCAA 3’ | | Forward primer for constructing translational fusion |
| *fur’-‘lacZ* rev | 5’ CTCCTCGGATCCGCGGTATTGTTATCAGTCATGCGGAA 3’ | | Reverse primer for constructing translational fusion |
| *fur* conf fwd | 5’ AGGCGTGGCAATTCTATAATGATACG 3’ | | Forward primer for confirming *fur* mutant |
| *fur* conf rev | 5’ CAAATAAGTGAGAGCTGTAACTCTCG 3’ | | Reverse primer for confirming *fur* mutant |
| LPF-12 | 5’ TTGTCGGTGAACGCTCTCCT 3’ | | pLFT sequencing primer |
| *pgaC* conf fwd | 5’ GGTTGTGGCAACATAATCAACCA 3’ | | Forward primer for confirming *pgaC* mutant |
| *pgaC* conf rev | 5’ CCCGACAATTTTCTGCATAACCA 3’ | | Reverse primer for confirming *pgaC* mutant |
| *ryhB*R | 5’ GCGATCAGGAAGACCCTCG 3’ | | Forward primer for *ryhB* northern probe |
| *ryhB*T7 | 5’ GTAATACGACTCACTATAGAAAAGCCAGCACCCGGCTG 3’ | | Reverse primer for *ryhB* northern probe |
| *sufA’-‘lacZ* fwd | 5’ GAGGAGCTGCAGCGTGCATTGATCCTGACTTAATGC 3’ | | Forward primer for constructing translational fusion |
| *sufA’-‘lacZ* rev | 5’ CTCCTCGGATCCGTTCCTGAATGCATGTCCATCG 3’ | | Reverse primer for constructing translational fusion |
| T7 *ftnB* For | 5’ GCTAATACGACTCACTATAGGGATCATCGTCAGAATTGACTCC 3’ | | Forward primer for RNA transcription in footprint |
| *ftnB* Rev | 5’ GGATCCTTGAGAAGCATTCCAGCGGTTGC 3’ | | Reverse primer for RNA transcription in footprint |
| T7 *ftnB* PstI For | 5’CGAGCCTGCAGTAATACGACTCACTATAGGGATCATCGTCAGAATTGACTCCACG 3’ | | Forward primer for cloning *ftnB* under T7 promoter for PURExpress |
| *ftnB* BamHI Rev | 5’ CTCCTCGGATCCTTGAGAAGCATTCCAGCGGTTGC 3’ | | Reverse primer for cloning *ftnB* under T7 promoter for PURExpress |
| T7 *dps* PstI For | 5’GAGCCTGCAGTAATACGACTCACTATAGGGTTAATTACTGGGACATAACATCAAGAGG 3’ | | Forward primer for cloning *dps* under T7 promoter for PURExpress |
| *dps* BamHI Rev | 5’ CTCCTCGGATCCGCGGTACTCATAATTTCATATCCTCTTGA 3' | | Reverse primer for cloning *ftnB* under T7 promoter for PURExpress |
| T7 *bfr* PstI For | 5’CTGCCTGCAGTAATACGACTCACTATAGGGTGGAAGCGAAGGAGTCAAAAAATG 3’ | | Forward primer for cloning *dps* under T7 promoter for PURExpress |
| *bfr* BamHI Rev | 5’ CTCCTCGGATCCACAAGCTCATTTCCCAACAGTTTGTTGAG 3’ | | Reverse primer for cloning *ftnB* under T7 promoter for PURExpress |

List of primers used in this study.

*Primers were purchased from Integrated DNA Technologies Inc., Coralville, Iowa.
